# Integrated inflammatory signaling landscape response after delivering Elovanoid free-fatty-acid precursors leading to experimental stroke neuroprotection

**DOI:** 10.1038/s41598-023-42126-w

**Published:** 2023-09-22

**Authors:** Madigan M. Reid, Ludmila Belayev, Larissa Khoutorova, Pranab K. Mukherjee, Andre Obenaus, Kierany Shelvin, Stacey Knowles, Sung-Ha Hong, Nicolas G. Bazan

**Affiliations:** 1 Neuroscience Center of Excellence, School of Medicine, Louisiana State University Health New Orleans, 2020 Gravier Street, Suite D, New Orleans, LA 70112 USA; 2 Department of Pediatrics, School of Medicine, University of California Irvine, Irvine, CA USA; 3 Present Address: UT Health, McGovern Medical School, University of Texas Health Sciences Center at Houston, Houston, USA

**Keywords:** Molecular biology, Neuroscience

## Abstract

Despite efforts to identify modulatory neuroprotective mechanisms of damaging ischemic stroke cascade signaling, a void remains on an effective potential therapeutic. The present study defines neuroprotection by very long-chain polyunsaturated fatty acid (VLC-PUFA) Elovanoid (ELV) precursors C-32:6 and C-34:6 delivered intranasally following experimental ischemic stroke. We demonstrate that these precursors improved neurological deficit, decreased T2WI lesion volume, and increased SMI-71 positive blood vessels and NeuN positive neurons, indicating blood–brain barrier (BBB) protection and neurogenesis modulated by the free fatty acids (FFAs) C-32:6 and C-34:6. Gene expression revealed increased anti-inflammatory and pro-homeostatic genes and decreases in expression of pro-inflammatory genes in the subcortex. Additionally, the FFAs elicit a comprehensive downregulation of inflammatory microglia/monocyte-derived macrophages and astrocyte-associated genes in the subcortical region. Functional analysis reveals inhibition of immune-related pathways and production of upstream molecules related to detrimental signaling events in post-stroke acute and subacute phases.

## Introduction

Ischemic stroke is a major burden to the worldwide healthcare system, with more than 800,000 cases annually in the United States, being the fifth leading cause of death and permanent disability^1^. The only FDA-approved therapy for stroke is IV administration of tissue plasminogen activator (tPA); however, it is available to patients that present within 4.5 h of stroke onset, and only 5–8% of patients qualify for this therapy^2^. The recent EXTEND trial demonstrated that this could be extended up to 9 h after stroke onset guided by CT or MRI perfusion images, therefore allowing more patients eligible to receive tPA beyond the 4.5-h time window^3,4^. Additionally, tPA fails to address the inflammatory and innate immune response associated with ischemia–reperfusion damage^5^. Despite active stroke research, the improvements in mechanical thrombectomy treatments have shown modest favorable clinical outcomes.

Ischemic stroke is a complex disorder with multifactorial pathophysiology that undergoes acute, subacute, and chronic phases. In the acute phase, ATP depletion and energy failure damaged cell membranes and organelles, releasing ROS and pro-inflammatory factors, including those engaged in the acute immune response. Reperfusion triggers an exacerbated ROS production, leading to uncompensated oxidative stress and endothelial cell injury. The influx of calcium ions activates cell signaling pathways, producing pro-inflammatory cytokines and chemokines. Additionally, ischemia activates immune cells, such as neutrophils and macrophages, which release pro-inflammatory cytokines and chemokines, exacerbating tissue damage and prolonging the inflammatory response^6^.

In the subacute stroke phase, edema evolves, which could increase intracranial pressure, and apoptosis is evident due to caspase activation, leading to neurons and other cells’ death. Also, the undergoing events that contribute to exacerbating brain damage are neuroinflammation, recruitment of immune cells that produce pro-inflammatory cytokines and chemokines, and the release of glutamate that activates excitotoxicity and contributes to neuronal damage^7,8^.

In the chronic phase of ischemic stroke, tissue repair, and functional recovery may involve complex and ununderstood events. Anti-inflammatory cytokines such as interleukin (IL)-10 and transforming growth factor-beta (TGF-β) are released, which helps dampen the inflammatory response; angiogenesis, neurogenesis, and synaptogenesis occur, contributing to the repair and improved neurological deficits^9^.

Multiple cells are involved, particularly microglia and astrocytes, which are activated within hours, producing cytokines and chemokines and fostering infiltration of leukocytes^10^. Secretion of cytokines and matrix metalloproteinases (MMPs) induces expression of cell adhesion molecules and enables immune cell infiltration^11^.

Microglia, the resident immune cells of the central nervous system (CNS), are rapidly activated after stroke^12^, displaying a phenotypic switch from a surveillant to a reactive state that is a pro-inflammatory (M1) and anti-inflammatory (M2) microglia. M1 microglia release pro-inflammatory cytokines, such as tumor necrosis factor-alpha (TNF-α), IL-1β, and IL-6, exacerbating the inflammatory response^13^. In contrast, M2 microglia IL-10 and TGF-β promote neuroprotection and tissue repair^14^. Following the acute phase of stroke, microglia participate in the recovery. M2 microglia promote neurogenesis, angiogenesis, and synaptic plasticity, facilitating recovery^15^. However, the M1 and M2 microglia balance determines the outcome, as a shift towards a pro-inflammatory M1 phenotype may impede recovery^16^.

Astrocytes are also involved in ischemic stroke^17^, exerting neuroprotection, sustaining homeostasis, transitioning to reactive astroglia (reactive gliosis), and migrating to the injury site. Thus, astrocytes provide neuronal support and protection. Reactive astrocytes display upregulated GFAP expression and morphological changes, secreting growth factors, cytokines, and extracellular matrix proteins, which promote cell survival, axonal growth, and synaptogenesis^18^. However, reactive astrocytes can also contribute to the pathogenesis of ischemic stroke by producing pro-inflammatory cytokines and ROS^19^. These cells can also contribute to the glial scar formation, a dense extracellular matrix that forms around the injury site and inhibits axonal regeneration^20^. Notwithstanding, there are potential benefits of targeting astrocyte-mediated processes in ischemic stroke. For example, modulating astrocytic glutamate transporters’ activity helps reduce excitotoxicity and improve neuronal survival after ischemic injury^21^. Other approaches include promoting the release of neurotrophic factors from reactive astrocytes or modulating the glial scar formation to enhance axonal regeneration^22^. This eventually results in the formation of a glial scar, which serves as a demarcation between the ischemic core and the surrounding penumbra.

DHA (omega-3 fatty acid)-derived free fatty acids (FFAs) are categorized as hormones because of their potential bioactivity exerted through G-protein coupled receptors (GPCRs). Due to the potent inflammatory modulatory activity of omega-3 FFAs, GPR120 is a receptor that mediates anti-inflammatory responses^23^. This receptor senses long-chain fatty acids eliciting anti-inflammatory effects through association with β-arrestin, which inhibits transforming growth factor-β-activated kinase and NLRP3 inflammasome activity. The NLRP3 inflammasome is a crucial component of the innate immune system, playing a role in ischemic stroke. It is a multi-protein complex that regulates the activation of caspase-1, leading to the production of pro-inflammatory cytokines, such as IL-1β and IL-18, contributing to the pathogenesis of ischemic stroke^24^. NLRP3 deficiency reduces infarct size, brain edema, and neurological deficits, supporting the role of the NLRP3 inflammasome in ischemic stroke pathogenesis^25^.

Elovanoids (ELVs) are novel pro-homeostatic neuroprotective lipid mediators derived from free very-long-chain polyunsaturated fatty acids (VLC-PUFAs)^26,27^. The enzyme ELOVL4 (elongation of very long-chain fatty acids–4) is responsible for the conversion of DHA into VLC-PUFAs (≥ C28, n-3). ELVs counteract UOS, oxygen–glucose deprivation (OGD), N-methyl-d-aspartate (NMDA) induced excitotoxicity, or middle cerebral artery occlusion (MCAo)-induced ischemic stroke. Administration of ELVs intracerebroventricular following MCAo decreases lesion volume and leads to sustained improvement in neurologic scores^26^.

There is a void in understanding the triggers and modulators of astrocytes and microglia in stroke pathophysiology. Here, we aim to explore whether intranasal administration of FFA precursors of ELVs would be neuroprotective and beneficially modulate the plasticity of reactive astrocytes and microglia responses reflected in neuroinflammatory gene expression.

## Results

### Intranasal C-32:6 or C-34:6 improves neurologic function and reduces lesion volume following stroke

**Figure 1:**
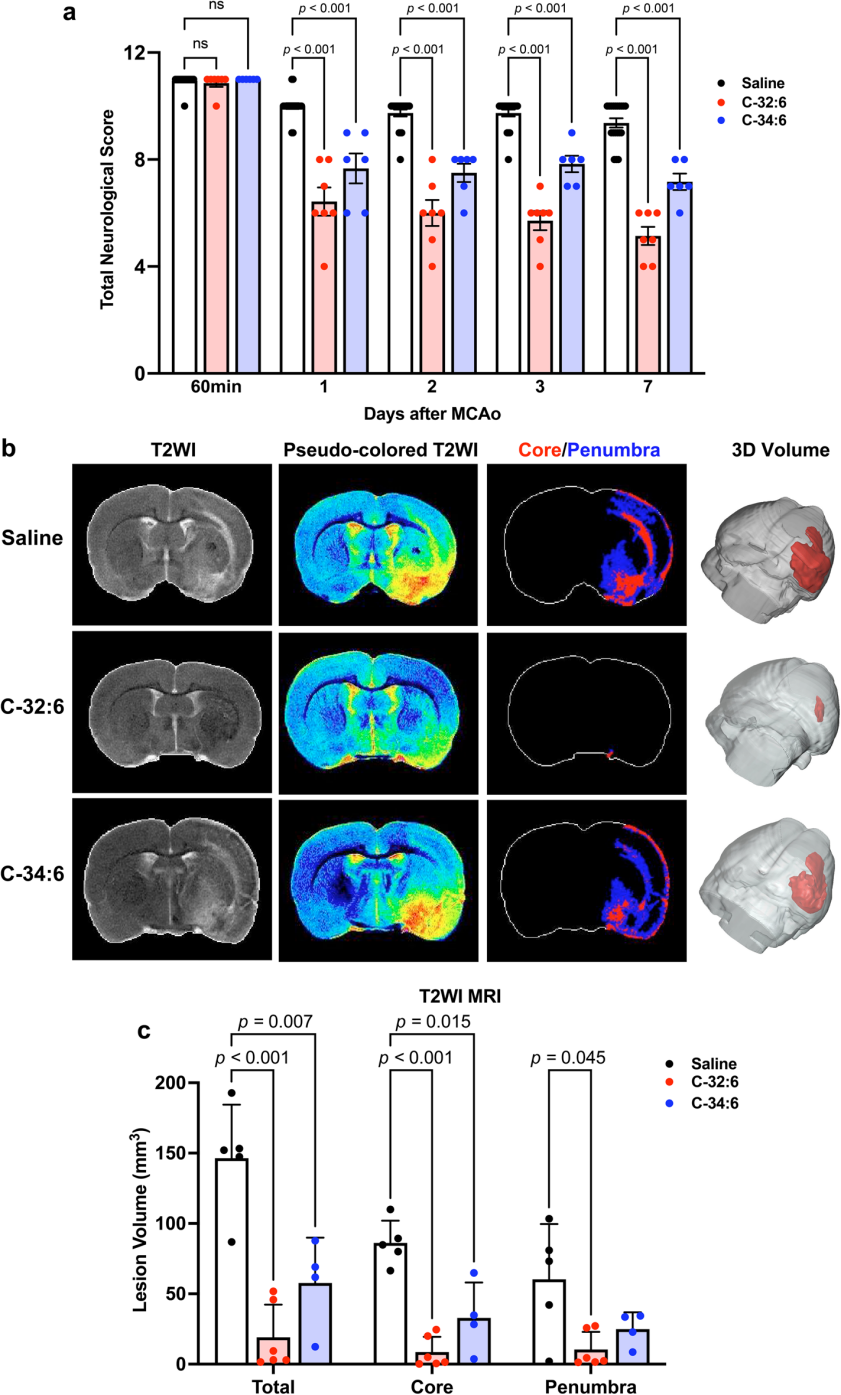
Intranasal administration of ELV FFA precursors promotes improvement of neurologic scores and decreases T2WI lesion volumes 7 days after MCAo. (**a**) Total neurologic score measured at 60 min, 1, 2, 3, and 7 days after MCAo. Values are means ± SEM; n = 6–12 per group. (**b**), Representative T2WI, pseudo colored T2WI, Core/Penumbra maps, and 3D reconstructed images from each treatment group are presented. (**c**), Volumetric measurements of total, core and penumbra lesions. Values shown are means ± SEM; n = 4–6 group.

The composite neurologic battery assessment demonstrated function recovery after intranasal treatment with C-32:6 and C-34:6 up to 7 days after stroke onset. The total neurologic score was improved by 36% or 23%, 38% or 23%, 41% or 20%, and 45% or 24% with C-32:6 or C-34:6 on days 1, 2, 3, and 7, respectively (Fig. 1a). Time course of recovery forelimb reactions showed improvements in tactile dorsal and lateral placing, and proprioceptive scores after stroke onset (Supplementary Fig. S1a-c). T2WI MRI analysis demonstrated that treatment with C-32:6 and C-34:6 decreased lesion volume (Fig. 1b,c). C-32:6 decreased total, core, and penumbra lesion volumes by 87%, 90%, and 83%, respectively. C-34:6 decreased total, core, and penumbra lesion volumes by 60%, 62%, and 59%, respectively. Representative bregma level images for all animals in the study demonstrate consistent reduction of lesion volume by C-32:6 and C-34:6 compared to vehicle (Supplementary Fig. S2a–c, Supplementary Table S1).

### C-32:6 and C-34:6 decrease microglial/monocyte-derived macrophages abundance, increase neuronal survival and protect blood–brain barrier integrity in the cortex and subcortex after ischemic stroke

**Figure 2:**
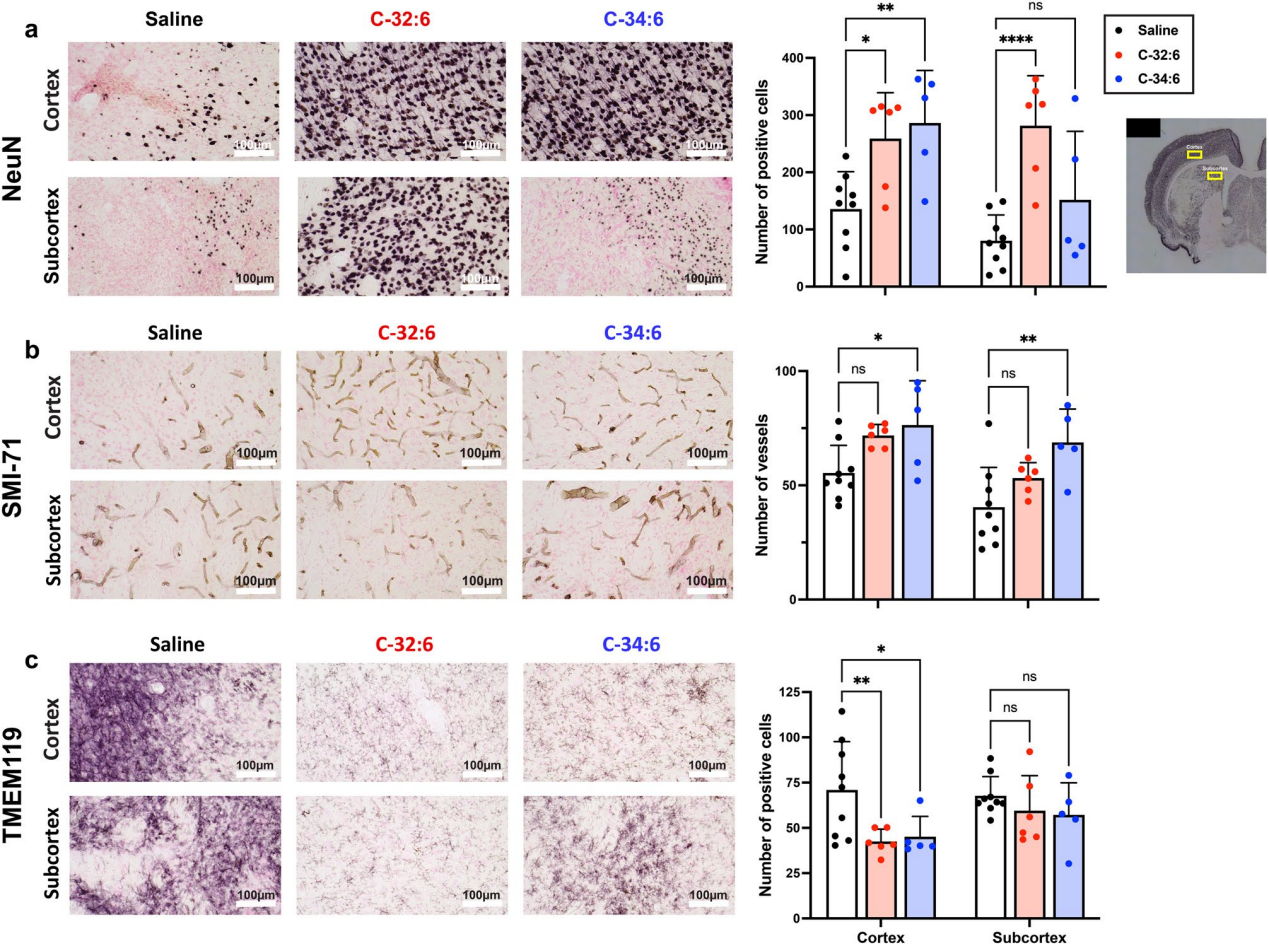
Cell count for Tmem119 positive cells, NeuN positive neurons, and SMI-71 positive vessels. (**a**), Representative images for NeuN positive neurons in the cortex and subcortex, cell count graphs show increased NeuN positive cells with all treatments in both regions. (**b**), Representative images for SMI-71-stained vessels for each group and vessel quantification graphs show increased SMI-71 positive vessels in both treatment groups compared to saline. (**c**), Images for Tmem119 positive cells in the cortex and subcortex and positive cell quantification graph showing decreased Tmem119 positive cells in the cortex with both treatments. Values shown are means ± SEM; n = 5–8 per group.

C-32:6 and C-34:6 intranasally increased the number of neurons in the cortex and subcortex following MCAo as evidenced by NeuN staining, with C-32:6 revealing more remarkable neuronal survival (Fig. 2a). SMI-71, an endothelial barrier antigen, is used as a marker to assess blood–brain barrier (BBB) integrity. Treatment with ELV precursors increased the quantity of SMI-71 positive vessels in the cortex and subcortex, inhibiting disruption of the neurovascular unit and likely facilitating neurogenesis and synaptogenesis (Fig. 2b). Under physiological conditions, TMEM119 is expressed in homeostatic microglia but not in other brain-resident cells nor in infiltrating macrophages; thus, it is commonly used as a marker to quantify microglia (Fig. 2c).

### C-32:6 and C-34:6 regulate gene expression involved in pro- and anti-inflammatory signaling after stroke

**Figure 3:**
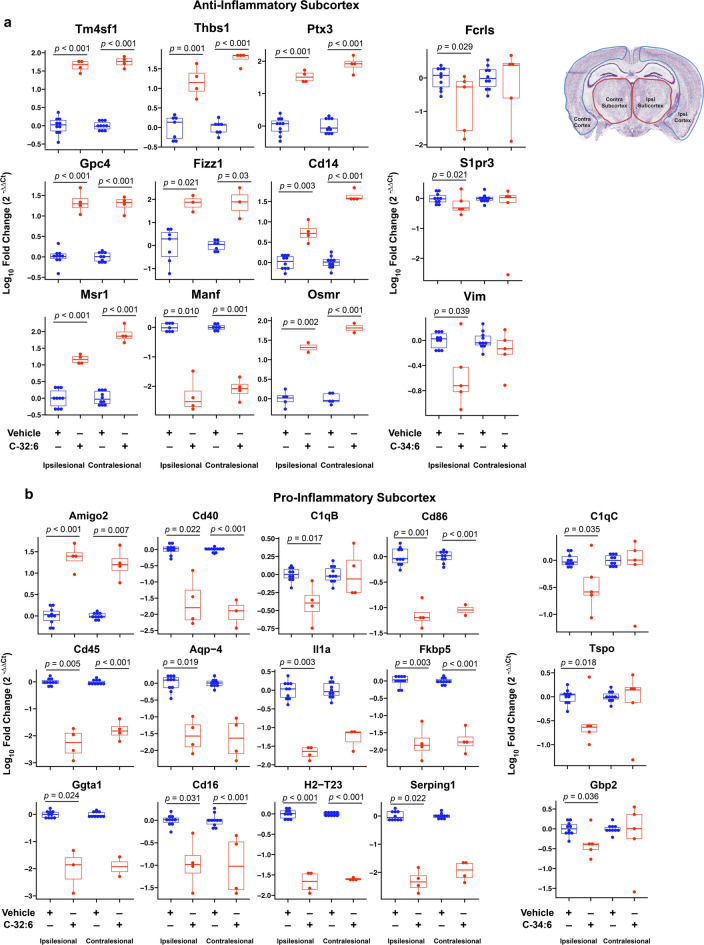
Free fatty acids C-32:6 and C-34:6 increase expression of anti-inflammatory genes and decrease expression of pro-inflammatory genes in the subcortex after ischemic stroke. Boxplots of log_10_ fold change values show significant differentially expressed anti-inflammatory (**a**) and pro-inflammatory (**b**) genes observed from C-32:6 and C-34:6 comparisons to vehicle in the subcortex. Statistics were computed from Wilcoxon-Mann–Whitney (WMW) analysis between each treatment and vehicle.

**Figure 4:**
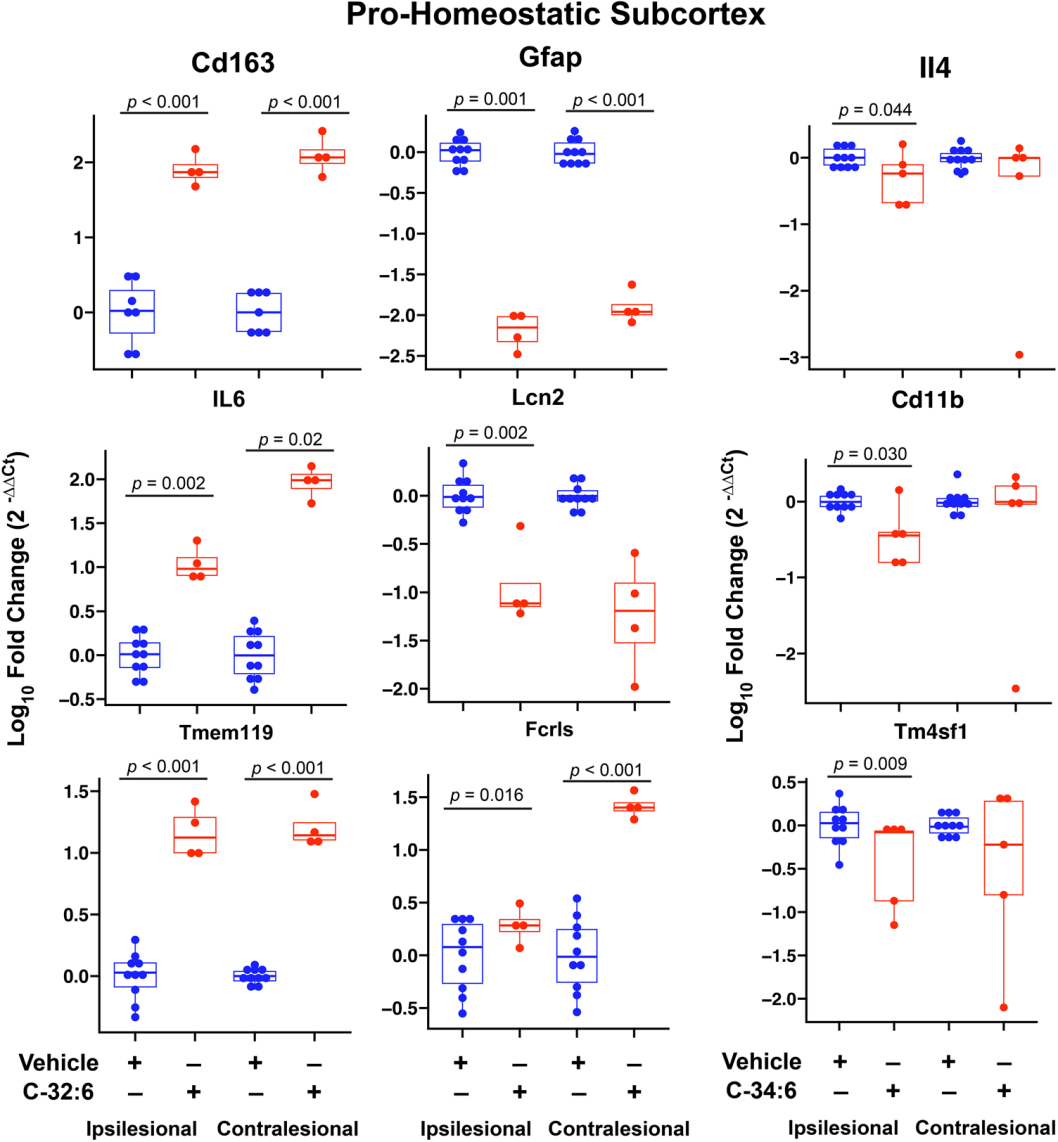
Modulation of pro-homeostatic gene expression is correlated with C-32:6 and C-34:6 administration after experimental ischemic stroke. Boxplots of log_10_ fold change values significant differentially expressed pro-homeostatic genes observed from C-32:6 and C-34:6 comparisons to vehicle in the subcortex. Statistics were computed from WMW analysis between each treatment and vehicle.

In brain samples harvested 3 days after stroke onset, changes were observed contra- and ipsilesionally, and modulation of expression was observed by both C-32:6 and C-34:6. The number of genes with significantly different expression levels compared to saline is: Contralesional Cortex C-32:6 (42) C-34:6 (6), Contralesional Subcortex C-32:6 (50) C-34:6 (3), Ipsilesional Cortex C-32:6 (11) C-34:6 (4), and Ipsilesional Subcortex C-32:6 (47) C-34:6 (14). Pro- and anti-inflammatory microglia/ monocyte-derived macrophages and astrocyte-associated gene expression are modulated by C-32:6 and C-34:6, as evidenced in heatmaps for cortex and subcortex regions for all treatment groups in ipsilesional and contralesional hemispheres (Supplementary Fig. S3a–d). Boxplots of log_10_ fold change values for the most significant pro- and anti-inflammatory genes for each treatment in the subcortex are shown in Fig. 3. Increases in anti-inflammatory genes *Tm4sf1, Thbs1, Ptx3, Gpc4, Fizz1, Cd14, MSr1,* and *Osmr* was modulated by C-32:6 (Fig. 3). C-32:6 also decreased pro-inflammatory genes *Amigo2, Cd40, C1qB, Cd86, Cd45, Aqp-4, Il1a, Fkbp5, Ggta1, Cd16, H2-T23,* and *Serping 1.* Heatmaps show the pro-homeostatic astrocyte and microglia/monocyte-derived macrophage-associated gene expression values for all genes in the cortex and subcortex for all treatment groups (Supplementary Fig. S4a,b). Boxplots of log_10_-fold change values for the most significant genes for C-32:6 and C-34:6 in the subcortex are depicted in Fig. 4. C-32:6 modulated increases in *Cd163*, *Il-6*, *Tmem119*, and *Fcrls* were observed.

### Functional gene expression and gene ontology reveal the targeting of macrophage signaling, innate immune response, and neuroinflammation gene networks by free fatty acids

**Figure 5:**
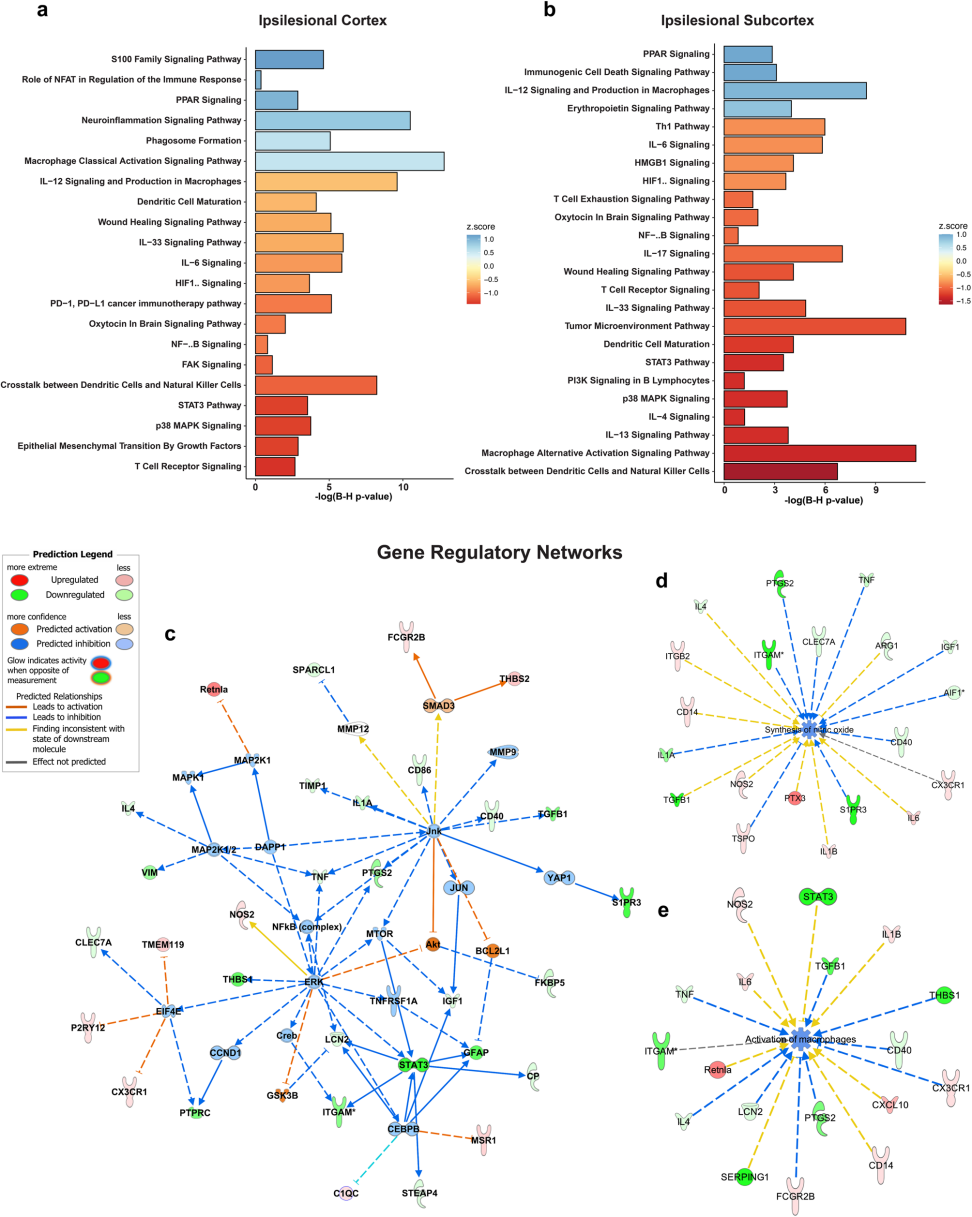
Ingenuity pathway analysis of C-32:6 gene expression demonstrates up and downregulation of canonical pathways relevant to stroke pathophysiology and gene networks reveal inhibition of genes and biological function detrimental in stroke progression. (**a**, **b**), Canonical pathway plots showing p-value and z-score computed from the ingenuity pathway analysis algorithm based on the uploaded gene set in the cortex (**a**) and in the subcortex (**b**). (**c**), Upstream causal network of genes shown to have direct causal relationship with downstream experimentally measured genes. (**d**, **e**), Inhibition of gene regulatory network for synthesis of nitric oxide (**d**) and regulatory network for activation of macrophages (**e**).

**Figure 6:**
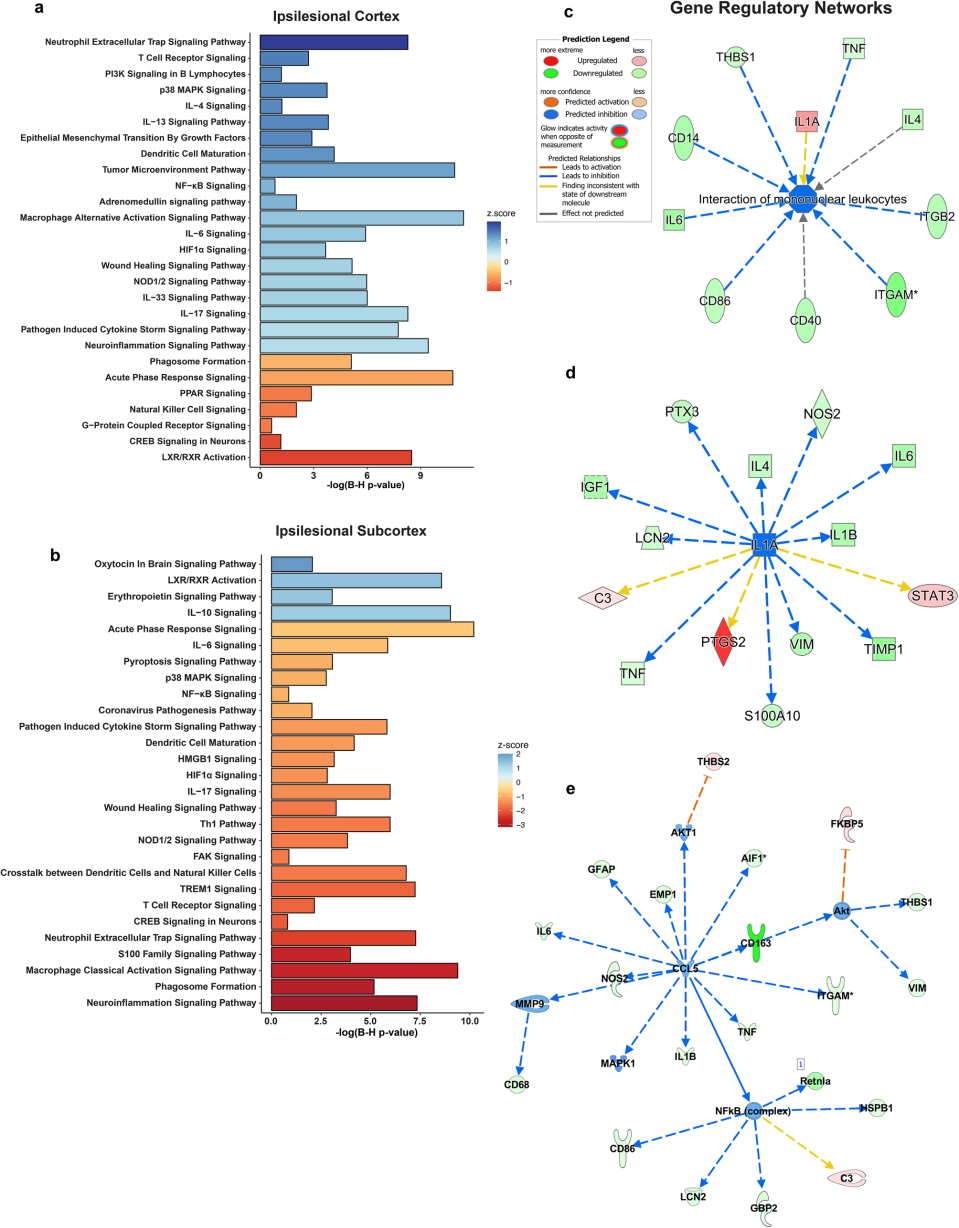
C-34:6 gene regulatory changes reveals predicted relationships with important molecular signaling pathways associated with stroke and inhibition of Il1a and interaction of mononuclear leukocytes. **(a–c**), Canonical pathway plots showing p-value and z-score computed from the ingenuity pathway analysis algorithm based on the uploaded gene set in the cortex (**a**) and in the subcortex (**b**), inhibition of the interaction of mononuclear leukocytes (**c**). (**d**), Predicted inhibition of Il1a gene regulatory network. (**e**), Upstream causal network of genes shown to have direct causal relationship with downstream experimentally measured genes.

Analysis of gene expression fold change ratios between C-32:6 and C-34:6 compared to the vehicle was performed with ingenuity pathway analysis. The top canonical pathways inhibited or activated by C-32:6 in the ipsilesional cortex and subcortex are presented in Fig. 5a,b. Analysis to determine canonical pathways gene enrichment was also performed on the data from contralesional cortex and subcortex samples, with top pathways being shown in Supplementary Figs. S5a,b and S6a,b. C-32:6 is predicted to inhibit T cell receptor signaling, p38 MAPK signaling, *NFkB, HIF1a*, among others, compared to vehicle in the cortex, and activation of phagosome formation, classical macrophage activation, S100, and neuroinflammatory pathways. In the subcortex, there is a distinct pattern in the canonical pathways modulated by C-32:6, where the most significant gene expression changes were observed. Upstream causal network predicting relationships from measured gene expression is shown in Fig. 5c. Gene regulatory network analysis with IPA predicted inhibition of nitric oxide synthesis and activation of macrophages in the subcortex (Fig. 5d,e). In the cortex, C-32:6 was predicted to inhibit ROS production, immune response of macrophages, and apoptosis (Supplementary Fig. S8a–c). C-34:6 functional analysis in the cortex indicated upregulation of adaptive immune response pathways, growth factor signaling, and wound healing pathway, among others, and the most significant inhibition of acute phase response signaling and PPAR signaling (Fig. 6a). In the subcortex, a larger number of pathways were inhibited, including IL-6, Acute phase response, Pyroptosis, HMGB1, HIF1α, and Neuroinflammation pathways (Fig. 6b). Gene regulatory networks for predicted the inhibition of the interaction of mononuclear leukocytes, IL-1α, and causal upstream regulatory genes *CCL5, Akt*, and *NFκB* (complex) in the subcortex are shown in Fig. 6c–e. In the cortex, gene regulatory networks show that immune responses of macrophages, leukocytes, and phagocytes are predicted to be inhibited by C-34:6, and survival of neural cells is activated (Supplementary Fig. S9a–d).

**Figure 7:**
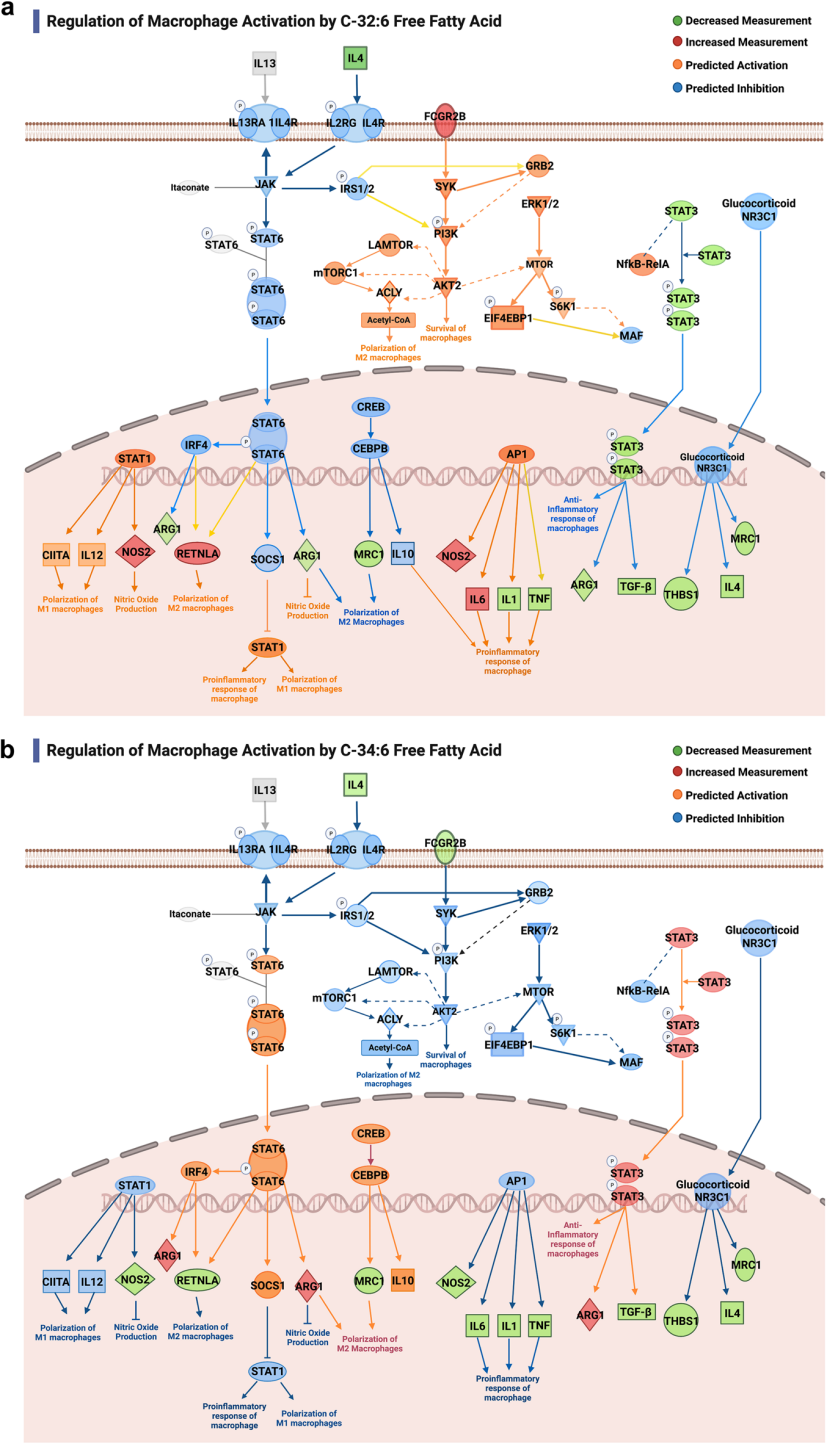
Macrophage activation pathway regulation in the subcortex by free fatty acids C-32:6 and C-34:6 demonstrating differences in bioactivity. (**a**), Differentially expressed genes from C-32:6 in the dataset are depicted in green and red for decreased and increased measurement, respectively; predicted relationships in the pathway are shown in orange and blue for activation and inhibition. (**b**), Shows regulation of the pathway by FFA C-34:6.

C-32:6 and C-34:6 showed macrophage activation canonical pathway changes differentially regulating upstream regulators affecting downstream signaling outcomes (Fig. 7a,b, Supplementary Tables S2–S3). C-32:6, most notably, is predicted to activate the polarization of M2 macrophages and promote macrophage survival from *Retnla (Fizz1)* and *PI3K/Akt*. However, C-34:6 demonstrated more beneficial regulation of this pathway, inhibiting the pro-inflammatory response of macrophages through predicted and measured regulation of *Stat1, Stat3, PI3K/Akt,* and *AP1* upstream.

Gene ontology analysis of the top upregulated and downregulated genes in the subcortex of rats treated with C-32:6 elucidated different biological functions enriched with these genes compared to what was observed from IPA analysis. Downregulation of genes (*Manf, Itgam, Serping1, Ptprc, Tnf, Timp1, Il1a,* and *Tgfb1*) by C-32:6 in the subcortex were enriched in GO terms associated with cytokine activity, complement binding, endopeptidase inhibitor activity, copper ion binding, and others (Supplementary Fig. S7a,b). Upregulated genes (*Retnlb* or *Fizz1, Hmgb1, Thbs1, Cx3cr1, Il1b, Il6, Osmr,* and *Cxcl1*0) upon treatment with C-32:6 show gene enrichment in GO terms for cytokine receptor binding, protein-lipid complex binding, scavenger receptor activity, integrin binding, and others shown in Supplementary Fig. S7c,d.

## Discussion

We show that intranasally administered FFA ELV precursors improved neurological deficit, decreased lesion volumes, and maintained neuronal and blood–brain barrier (BBB) integrity after experimental ischemic stroke. Alterations in genes that participate in endogenous reparative processes promoting recovery after stroke are thought to drive this neuroprotection. We showed that the FFA targeted gene expression, through HT-qPCR, of anti- and pro-inflammatory microglia/monocyte-derived macrophages and astrocytes. Recent advances in endovascular intervention and enzymatic clot removal have facilitated reperfusion after ischemic stroke, yet there are still no efficacious therapies to prevent and repair damage, as well as regulate ischemia–reperfusion injury-associated inflammatory response involving pro- and anti-inflammatory microglia/monocyte-derived macrophages and astrocytes.

Ischemic stroke can impair sensorimotor and cognitive functions, and more than 50% of patients present sustained physical disabilities. Thus, assessment of neurobehavioral recovery is critical to determine neuroprotective mechanisms in pre-clinical models. Using a composite neurologic battery demonstrated improved neurological deficit with FFAs C-32:6 and C-34:6 up to 7 days after stroke onset.

Following stroke onset, the inflammatory response comprises the activation of microglia/monocyte-derived macrophages and astrocytes and infiltration of circulating cells due to BBB disruption, such as granulocytes, neutrophils, monocytes, macrophages, and T cells, worsening tissue damage^28^. The integrity of blood vessels sustains neurogenesis and synaptogenesis, contributing to improved neurological deficits. Our data uncovered that BBB integrity is protected by the FFAs, using SMI-71 as a marker, which binds to endothelial barrier antigen (EBA), a protein expressed by endothelial cells in the BBB of cortical and subcortical regions. We observed that neuronal loss was decreased in animals treated with C-32:6 and C-34:6 in the cortex and subcortex, indicating that their bioactivity contributes to neuronal survival. Additionally, T2WI MRI analysis revealed reductions in core and penumbra lesion volumes, confirming protection by C-32:6 and C-34:6 FFAs in experimental ischemic tissue.

Modulation of gene expression by FFAs C-32:6 and C-34:6 was much greater in the subcortical than in cortical regions. Additionally, C-32:6 modulated the expression of a larger number of genes with greater significance. Differences in gene expression were also observed bilaterally rather than isolated to the ipsilesional side. Recently, RNA-seq experiments have demonstrated that changes in expression levels of genes occur in both hemispheres after ischemic stroke, most notably inflammatory and immune signaling-associated genes^29^. The microglia/monocyte-derived macrophages and astrocyte-specific genes investigated were categorized by functional significance in inflammatory signaling. However, the previous understanding of microglia subtypes M0, M1, and M2 is changing due to an increased understanding of their molecular function in brain homeostasis. In response to insults, microglia adapt their function to restore brain homeostasis^30^. Microglia can undergo a phenotypic transformation during sustained activation to disease-associated microglia (DAM) associated with neurodegeneration. This transformation is a protective response to prevent neuronal damage^31^. Our investigation of the effect of novel FFAs C-32:6 and C-34:6 on pro- and anti-inflammatory astrocyte and microglia/monocyte-derived macrophage-associated genes showed regulation of multiple genes that could provide a mechanistic explanation for their protective bioactivity.

Anti-inflammatory marker genes for protective microglia and astrocyte phenotypes *Tm4sf1, Thbs1, Ptx3, Gpc4, Fizz1, Cd14, Msr1, Manf*, and *Osmr* were upregulated with C-32:6 and genes *Fcrls, S1pr3,* and *Vim* were downregulated with C-34:6. Decreased expression of pro-inflammatory genes, which are associated with neurodegenerative disease or astrocyte and microglia phenotypes *Cd40, C1qB, Cd68, Cd45, Aqp-4, Il1α, Fkbp5, Ggta1, Cd16, H2-T23*, and *Serping1*, was observed following C-32:6 treatment and *C1qC, Tspo*, and *Gbp2* with FFA C-34:6. Upregulation of anti-inflammatory associated genes by C-32:6 in the subcortex indicates a potential bioactive mechanism underlying the observed neuroprotection revealed by neurobehavioral assessment and MRI analysis*. Osmr*, an oncostatin m receptor, has been demonstrated to be neuroprotective in vivo and in vitro ischemic stroke through recruitment of OSMRβ and that overexpression of OSMRβ is cerebroprotective, though the exact mechanism is not yet known^32^. The pro-survival effect of Mesencephalic astrocyte-derived neurotrophic factor (*Manf*) has been shown in multiple disease states such as diabetes, stroke, and neurodegeneration regulating neurogenesis and inflammation^33^. In the context of stroke, *Gpc4*, a member of the glypican family, has been shown to exert neuroprotective effects promoting neuronal survival, angiogenesis, neurogenesis, and regulation of synaptic plasticity through regulation of various growth factors such as fibroblast growth factors (FGFs), Wnts, and bone morphogenic proteins (BMPs)^34^. Another gene that we observed to be upregulated by C-32:6 is *Ptx3*, which is expressed in neurons and glia in response to cerebral ischemia and induced by IL-1; it may be a critical effector of brain edema and glial scar formation aiding in post-stroke recovery^35^.

In contrast to C-32:6, C-34:6 primarily downregulated gene expression in the subcortex and cortex regions; thus, pro-inflammatory genes whose expression was significantly decreased may provide more substantial insight into the neuroprotective effects. Reduced expression of *Gbp2* modulated by C-34:6 may indicate pro-survival and anti-apoptotic mechanistic targets. *Gbp2* protein levels increase after brain injury, and overexpression in neuronal cell types has been shown to aggravate neuronal apoptosis^36^. Additionally, decreased expression of *C1qC* was observed, which is involved in complement cascade activation. *C1q* is thought to play a role in the acute phase of stroke, and inhibition of *C1q* may be beneficial in attenuating ischemic brain injury^37^. *Tspo* downregulation mediated by C-34:6 could elucidate a larger mechanism, as recently it has been shown the knockdown of *Tspo* repressed NLRP3 inflammasome activation in OGD/R treated neurons as well as attenuated apoptosis indicating that *Tspo* may play a critical role in ischemia–reperfusion injury^38^.

FFA C-32:6 was also observed to upregulate a small number of pro-homeostatic genes; however, C-34:6 was not observed to have the same effect. Most notably, upregulation of *Cd163* indicated that C-32:6 promotes the resolution of inflammation and helps to transition to the recovery phase. Cd163 is an anti-inflammatory Hb scavenger receptor induced by anti-inflammatory cytokines (IL-6 and IL-10), and expression is repressed in the presence of inflammatory stimuli (IL-4, TNFα, IFN-γ, and LPS)^39^.

The mechanistic insights provided by this study suggest that through upstream effects, the free fatty acids act on genes which, when increased or decreased, will aid in moving the sum of the signaling events toward the chronic recovery phase by regulating endogenous repair processes. The overall gene expression changes observed from free fatty acid C-32:6 indicate that its modulatory effects promote the biological activity of astrocytes, microglia/monocyte-derived macrophages, and cerebrovascular cells involved in plasticity. C-34:6 did not regulate gene expression profiles as notably as C-32:6; however, the observed neuroprotection provided does correlate with the changes it induced.

## Conclusion

Recent advances in endovascular intervention and enzymatic clot removal have facilitated reperfusion after ischemic stroke, yet there are still unmet needs to prevent and repair damage. The effect of the FFA on the damaging inflammatory signaling cascade would complement therapeutic protection.

## Limitations of research and alternatives

Increasing knowledge regarding different cell phenotypes during the pathogenesis of stroke provides critical information but also indicates that many of the genes previously believed to serve one function participate in post-stroke signaling in multifaceted ways. Literature often reports contradictory findings regarding the role of these genes; thus, the interpretation of results must be cautious. In this study, we investigated 76 genes of interest; however, based on the results, additional genes have been identified, which will be investigated in future studies, as well as the production of protein end products from the most differentially expressed genes. Additionally, the effect of lipid mediators in ischemic stroke was not investigated in female or aged rats, which will be performed in the future.

## Methods

### Animals and surgical preparation

All studies were approved by the Institutional Animal Care and Use Committee of the Louisiana State University Health Sciences Center (IACUC protocol number 3778). Animal care was performed in accordance with relevant institutional and national guidelines and regulations of the National Institutes of Health (NIH). The studies were performed following the ARRIVE Guidelines. Male Sprague–Dawley rats (270-350g) from Charles River Laboratories (Wilmington, MA) were used in all studies. Atropine sulfate (0.5 mg/kg, i.p.) was injected 10 min before anesthesia. Anesthesia was induced with 3% isoflurane in a mixture of 70% nitrous oxide and 30% oxygen. All rats were orally intubated and mechanically ventilated. During ventilation, the animals were paralyzed with pancuronium bromide (0.6 mg/kg, i.p.). Catheters were inserted into the right femoral artery and vein for blood sampling. Arterial blood gas analysis, plasma glucose, hematocrit, and arterial blood pressure were measured before and during the surgical procedure. Rectal (CMA/150 Temperature Controller, CMA/Microdialysis AB, Stockholm, Sweden) and cranial (temporalis muscle; Omega Engineering, Stamford, CT) temperatures were maintained at 36–37 °C before, during, and after MCAo. Rectal temperature and body weight were monitored daily during the survival period.

### Transient middle cerebral artery occlusion

Rats underwent 2 h of right middle cerebral artery occlusion (MCAo) by the intraluminal-suture occlusion model, in which a nylon filament coated in poly-L-lysine is introduced into the middle cerebral artery, as previously described^40^. A 3–0 monofilament nylon suture was passed via the proximal ECA into the internal carotid artery and, thence, into the MCA, a distance of 20–22 mm from the carotid bifurcation according to the animal’s weight. The nylon filament is coated in poly-L-lysine prior to insertion to enhance adhesion to the surrounding endothelium^40^. After 2 h of MCAo, rats were re-anesthetized with the same anesthetic combination, intraluminal sutures were removed, and the animals were allowed to recover with free access to food and water.

### Assessment of functional neurological outcome

The experiments were performed between 8:00 a.m. and 4:00 p.m. by researchers blinded to the treatment groups. Rats underwent neurobehavioral testing before MCAo and on days 1, 2, 3, and 7 after MCAo. All tests were performed by an investigator blinded to the treatment groups. A neurological battery consisted of two components: (1) a postural reflex test, designed to examine forelimb and upper-body posture in response to tail-suspension and lateral displacement, regarded as being sensitive to both cortical and striatal lesions; and (2) an elicited forelimb placing test, which examines sensorimotor integration by assessing placing reactions to visual, tactile, and proprioceptive stimuli. The total neurologic score is graded on a scale of 0 (no deficit) to 12 (maximal deficit), as previously described^40^.

### Treatments and experimental protocols

#### Neurobehavior, T2WI MRI, and Histopathology

C-32:6 or C-34:6 (Cayman Chemical Co, Ann Arbor, Michigan, USA) were dissolved in 0.9% saline and administered (IN) 3, 24, and 48 h after stroke onset. Vehicle (0.9% saline) was administered at 3, 24, and 48 h, and animals were sacrificed 7 days after stroke onset. All experiments were done by researchers blinded to the treatment groups. For these experiments, an n = 6–12 per group for neurologic evaluation, n = 4–6 for T2WI MRI analysis, and n = 5–9 for histopathology.

#### RT-qPCR Gene Expression

A separate animal cohort was assigned to 3 groups: Vehicle, C-32:6, or C-34:6 (20 μg/20 μl) administered at 3, 24, and 48 h after the onset of MCAo. The behavioral evaluation was conducted, followed by brain sampling of the ipsilesional and contralesional cortex and subcortex at 3 days. For gene expression experiments, n = 4–10 per group.

### MRI acquisition and analysis of total lesion, core, and penumbra volumes

High-resolution ex vivo MRI was conducted on 4% paraformaldehyde-fixed brains on day 7 using a 9.4 T or 11.7 T Bruker Advance (Bruker Biospin, Billerica, MA, USA) with the following parameters: TR/TE 2400/10.6, 10 evenly spaced echoes 10 ms apart, matrix 256^2^, and 20 slices 1 mm thick. T2-weighted imaging (T2WI) and T2 relaxation maps were computed as we previously described^41^. Hierarchical Region Splitting (HRS) was used to automatically identify core and penumbra volumes (total lesion = core + penumbra) from T2 relaxation maps^41^ (implemented in Matlab) and has been previously validated by using perfusion-weighted imaging (PWI)/diffusion-weighted imaging (DWI)^41^. The penumbra was defined using T2 values (ms) between normal-appearing brain tissue and the ischemic core. T2 values (ms) and volumes were extracted from normal-appearing penumbra and core brain tissue and were summarized per group.

### Histopathology and immunostaining

#### Neurohistology embedding, sectioning & staining

Following MRI imaging, 4% paraformaldehyde-fixes brains were sent to NeuroScience Associates. Brains were examined and treated overnight with 20% glycerol and 2% dimethylsulfoxide to prevent freeze-artifacts. The specimens were then embedded in a gelatin matrix using MultiBrain® Technology (NeuroScience Associates, Knoxville, TN). The blocks were rapidly frozen after curing by immersion in 2-methylbutane chilled with crushed dry ice and mounted on a freezing stage of an AO 860 sliding microtome. The MultiBrain® blocks were sectioned in coronally with the desired micrometer (µ) setting on the microtome. All sections were cut through the entire length of the specimen segment and collected sequentially into a series of 24 containers. All containers contained Antigen Preserve solution (50% PBS pH7.0, 50% Ethylene Glycol, 1% Polyvinyl Pyrrolidone); no sections were discarded.

#### Immunohistochemistry

Free-floating sections were stained to detect specific markers Tmem119, SMI-71, and NeuN. For Tmem119 detection, sections were incubated with Tmem119 at a concentration of 1:50,000 (Cat#400211, Synaptic Systems). For SMI-71 detection sections were first incubated with SMI 71 IHC Stain at a dilution of 1:500 (Cat#836,04, BioLegend) and subsequently incubated with secondary antibody (Anti-Mouse Biotinylated, Cat#ba-2001, Vector) at a dilution of 1:1000. For NeuN detection, sections were initially stained with NeuN IHC Stain (Cat#ab104225, Abcam) at a dilution of 1:15,000 and subsequently stained with secondary antibody (Anti-Rabbit Biotinylated, Cat# ba-1000, Vector) at a dilution of 1:1000. All incubation solutions from the blocking serum onward use Tris-buffered saline (TBS) with Triton X-100 as the vehicle; all rinses are with TBS. After a hydrogen peroxide treatment and blocking serum, the sections were immunostained with the primary antibodies overnight at room temperature. Vehicle solutions contained Triton X-100 for permeabilization. A biotinylated secondary antibody (anti-IgG of host animal in which the primary antibody was produced) was applied following rinses. After further rinses, Vector Lab's ABC solution (avidin–biotin-HRP complex, Vector, Burlingame, CA) was applied. The sections were again rinsed and then treated with diaminobenzidine tetrahydrochloride (DAB) and hydrogen peroxide to create a visible reaction product. The sections were mounted on gelatin-coated glass slides and air-dried after further rinses. The slides were dehydrated in alcohol, cleared in xylene, and coverslipped.

### Imaging

NeuN, SMI-71, and Tmem119 stained brain sections at the bregma level − 0.3 mm were used to acquire bright-field images using the microscope (Leica DMI81, FlexiCam C1, Leica) with an × 20 magnification of the objective lens. The cortical and striatal images in the peri-infarct region were obtained as depicted (Fig. 2a). Image-J (NIH) was used to process and analyze the images. Obtained images were converted into binary images, followed by threshold adjustment. The number of NeuN-positive cells and SMI-71 labeled vessels were quantified by the Analyze Particles function. The population of TMEM119-positive activated microglia was measured by mean pixel intensity within the image field.

The pixel intensity in the image analyzed the population of TMEM119-positive microglia because of the difficulty/limitation in counting individual activated microglial soma by thickened processes and clustered activated microglia in the peri-infarct regions.

### HT-RT-qPCR

#### Primer selection and development

Seventy-six transcripts of differentially expressed genes following ischemic stroke were selected for analysis (Supplementary Table S4). Candidate genes were selected from a literature search to determine stroke-associated astrocyte and microglia/monocyte-derived macrophage subtype-specific genes that have been shown to be differentially expressed following stroke or play a role in stroke pathophysiology^42–46^.

#### Sample collection and RNA isolation

Brain samples of the anterior and posterior ipsilesional cortex and subcortex regions were harvested 3 days after stroke onset, flash-frozen in liquid nitrogen, and stored at − 80 °C until use. Tissue samples from each region were homogenized using TRIzol® reagent (Cat. #15596026l, Invitrogen, Thermo Fisher Scientific, Inc., Waltham, MA, USA) according to the manufacturer’s protocol. Total RNA was prepared using RNeasy columns (Cat. #74004, Qiagen, Valencia, CA). RNA quantity and purity were assessed using the NanoDrop One spectrophotometer (Thermo Scientific). Optimal purity of RNA was ensured by determination of the 260/280 adsorption ratio (values > 2.00).

#### Reverse transcription

One microgram of total RNA was reverse-transcribed per sample into first-strand complementary DNA (cDNA). cDNA samples were stored at − 20 °C for a maximum of 4 weeks.

#### Specific target amplification (STA)

A specific target gene amplification (STA) was conducted to ensure sufficient target gene templates for high-throughput qPCR. For STA, all target gene sequence-specific primer pairs were pooled and diluted with DNA suspension buffer to a final concentration of 500 nM (pooled primer mixture), and stock solutions were stored at − 20 °C. A 5 µL STA mix was prepared for each reaction, containing 2.5 µL 2 × PreAmp Master Mix, 0.5 µL of the 500 nM pooled primer mixture, 0.75 µL PCR-certified water, and 1.25 µL cDNA. PCR-certified water control (NTC-STA) and a non-reverse-transcribed RNA (NoRT) control were also included. STA was performed in a thermal cycler (Mastercycler® nexus, Eppendorf) using a temperature program consisting of an initial 10-min denaturation at 95 °C, 12 cycles of 15-s denaturation at 95 °C and 4-min annealing and elongation at 60 °C, and a final holding temperature of 4 °C. To eliminate unincorporated primers post-STA, samples were treated with exonuclease I (E. coli; Cat. #M0293S, New England Biolabs). A 2 µL exonuclease reaction mixture was added to the STA samples. Digestion with Exo I at 4 units/µL was carried out in a thermal cycler using a temperature program consisting of 40 min at 37 °C for digestion of unincorporated primers and dNTPs, 15 min at 80 °C for Exo I inactivation, and a final holding temperature of 4 °C. STA and Exo I-treated samples were then diluted tenfold with 43 µL TE buffer.

#### Preparation of samples and primers

Forward and reverse primers (100 µM) were diluted to 5 µM by adding 2.5 µL of each primer pair to 25 µL of 2 × Assay Loading Reagent and 22.5 µL of DNA suspension buffer. The primer reaction mix was stored at − 20 °C. For the sample mix, 2.25 µL of STA and Exo I-treated samples were mixed with 2.5 µL of 2 × SsoFast™ EvaGreen® Supermix with Low ROX and 0.25 µL of 20 × DNA Binding Dye Sample Loading Reagent (Cat. #1725211; Bio-Rad).

#### High-throughput RT-qPCR

HT RT-qPCR was run on the BioMark HD System, using 96 × 96 Fluidigm Dynamic Arrays (Cat. #BMK-M-96.96; Fluidigm, South San Francisco, CA). Preparation and loading of Fluidigm 96.96 Dynamic Array IFC (integrated fluidic circuit) were performed according to the manufacturer's instructions. Preparation of the 96.96 Dynamic Array IFC included the injection of 150 µL of a control line fluid into each chip accumulator with a syringe. The chip was then placed into the Juno and run with the prime 96.96 GE. After priming, the chip was loaded with samples, and the primer reaction was mixed within 1 h to reduce the pressure loss within the chip. Thus, 5 µL of each primer reaction mix and each sample were loaded into respective inlets. Samples and primer reaction mixes were loaded into the chip into the Juno and running the Load mix 96.96 GE script. The chip was transferred into the BioMark™ HD System, and qPCR and melting curve analysis were performed by running the following temperature program: 2400 s at 70 °C and 30 s at 60 °C, followed by a hot start for 60 s at 95 °C, 30 PCR cycles of 5 s at 96 °C for denaturation and 20 s at 60 °C for annealing and elongation. The melting curve analysis consisted of 3 s at 60 °C followed by heating up to 95 °C with a ramp rate of 1 °C/3 s.

#### RT-qPCR data analysis

Raw data were pre-processed with the Real-Time PCR analysis software v4.1.3(Fluidigm); unspecific values were deleted based on melting-curve analysis. The relative mRNA expression levels of predicted key genes were calculated using the 2^−ΔΔCT^ method, following normalization to the geometric mean of housekeeping genes:

#### Network establishment and analysis

Each brain region's dataset was built and integrated into the IPA system for in-depth analysis and data visualization. The network analysis algorithm utilized Fisher’s exact test with an enrichment score of P-values. Genes with known Ensembl gene IDs and their respective expression values were input into the software. In the Ingenuity Pathways Knowledge Base, every gene symbol was linked to its corresponding gene object. The software generated gene networks algorithmically based on their connectivity and assigned them a score. This score considers the number of focus genes within the network and the network's size, approximating the network’s relevance to the initial gene list. The identified networks are displayed as graphs illustrating the molecular connections between genes or gene products. The software also produces upstream causal networks, which indicate potential or predicted upstream relationships based on the input gene list and enrichment plots for the most significant or highest-scoring pathways containing genes from the uploaded dataset. The “path designer” module was employed to refine the network images. Canonical pathway enrichment data from IPA was exported, and histograms were created using RStudio 2021.09.0. IPA utilizes four distinct causal algorithms: (1) Upstream Regulator Analysis (URA) identifies potential regulators; (2) Mechanistic Networks (MN) enhance URA by linking regulators likely involved in the same signaling or causal mechanism within hypothesis networks; (3) Causal Network Analysis (CNA) expands upon URA by connecting upstream regulators to dataset molecules via paths with more than one link (e.g., through intermediate regulators), generating a comprehensive view of potential root causes for observed expression changes; and (4) Downstream Effects Analysis (DEA) applies URA principles to deduce and evaluate the influence on biological functions and diseases that are downstream of genes with altered expression in a dataset^47^. The networks identified are then presented as a graph indicating the molecular relationships between genes/gene products. The “path designer” module was used to polish the network images and graphs.

### Gene ontology enrichment analysis

The top upregulated and downregulated genes for C-32:6 and C-34:6 in the ipsilesional subcortex were analyzed with enrichGO in R using the cluster Profiler package assigning GO terms in which the selected genes are enriched. The analysis results were visualized using barplots, gene concept networks, and enrichment maps using DOSE and enrichplots.

### Statistical analysis

A priori power analysis was conducted using G*Power version 3.1.9.7 for sample size estimation based on Neurological data from a previous study^48^. With a significance criterion of α = 0.05 and power = 0.80, the minimum sample size needed with this effect size is N = 3 for the Wilcoxon-Mann–Whitney (WMW) test of the two groups. Thus, the obtained sample size of N = 4–10 is more than adequate. The same parameters for alpha and power were used for gene expression studies, and group means, and SD were determined from a pilot study using the same methodology described above. The minimum sample size needed with this effect size is N = 4 for Welch’s t-test of two groups. Statistical testing was performed using RStudio 2021.09.0. Shapiro’s test was used to assess normality. For statistical testing of ∆Ct values, normalized against the geometric mean of housekeeping genes, the conservative assumption of unequal variance was made. Welch's t-test was used for comparisons between treatment and control groups (Supplementary Tables S5–S8). Welch’s t-test analyzed parametric MRI data, and non-parametric neurological data was analyzed using the WMW test (Supplementary Tables S9–S12). Neurological and MRI data are presented as means ± SEM with individual points for biological replicates shown. Gene expression data for significant genes is presented in boxplots with replicates shown as individual points. Values which statistical tests determined *p* < 0.05 were considered significant.

### Supplementary Information

Supplementary Information.

## Data Availability

There are no restrictions on materials. All data are available in the main text or the supplementary materials.

## Code availability

This research did not involve developing or using any custom code, software, or algorithms. All data analyses were performed using standard, commercially available software and tools. Details on the software, tools, and their respective versions can be found in the Methods section of the manuscript. Please contact the corresponding author for any inquiries regarding the data analysis process.
