# Supplementary material for: Integrated inflammatory signaling landscape response after delivering Elovanoid free-fatty-acid precursors leading to experimental stroke neuroprotection
Source: Sci Rep. 2023 Sep 22;13:15841. doi: 10.1038/s41598-023-42126-w (PMC10516907; doi:10.1038/s41598-023-42126-w)
Supplement: Supplementary file 1 — Supplementary Information. [file 41598_2023_42126_MOESM1_ESM.pdf]

Supplementary Information

Integrated inflammatory signaling landscape response after delivering Elovanoid free-fatty-acid precursors leading to experimental stroke neuroprotection

Madigan M. Reid, BS[1], Ludmila Belayev, MD[1], Larissa Khoutorova, BS[1], Pranab K. Mukherjee, PhD[1], Andre Obenaus, PhD[3], Kierany Shelvin, MS[1], Stacey Knowles[1], Sung-Ha Hong, PhD[1,2], Nicolas G. Bazan, MD, PhD[1*]

[1]Neuroscience Center of Excellence, Louisiana State University Health New Orleans, 2020 Gravier Street, Suite D, New Orleans, USA.
[2]Present address: UT Health, University of Texas Health Sciences Center at Houston, McGovern Medical School, Houston, USA.
[3]Department of Pediatrics, School of Medicine, University of California Irvine, Irvine, CA, USA.

***Corresponding author:**
Nicolas G. Bazan, MD, PhD, Neuroscience Center of Excellence, Louisiana State University Health New Orleans, School of Medicine, Neuroscience Center of Excellence, 2020 Gravier Street, Suite D, New Orleans, LA 70112; Phone: +1 (504) 599-0832; E-mail: NBazan@lsuhsc.edu; ORCID ID: 0000-0002-9243-5444.

Contents

Supplementary Figs. S1-S9
Supplementary Tables S1-S12

SUPPLEMENTARY FIGURES

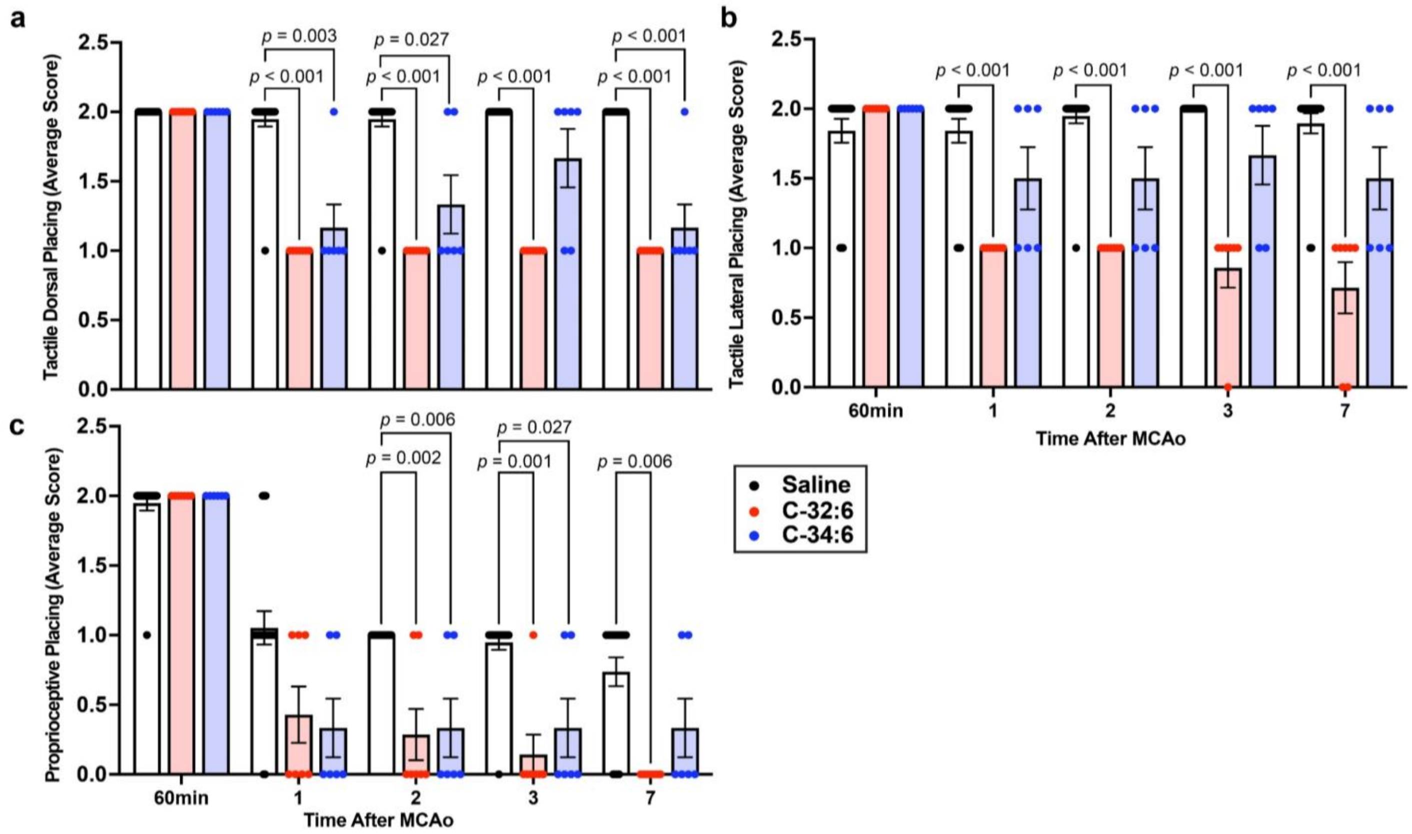

Supplementary Fig. S1: Individual neurologic test results for tactile dorsal placing (**a**), tactile lateral placing (**b**), and proprioceptive placing scores (**c**). Values are mean ± SEM, p-values shown from Wilcoxon Mann Whitney comparison between each treatment vs. vehicle.

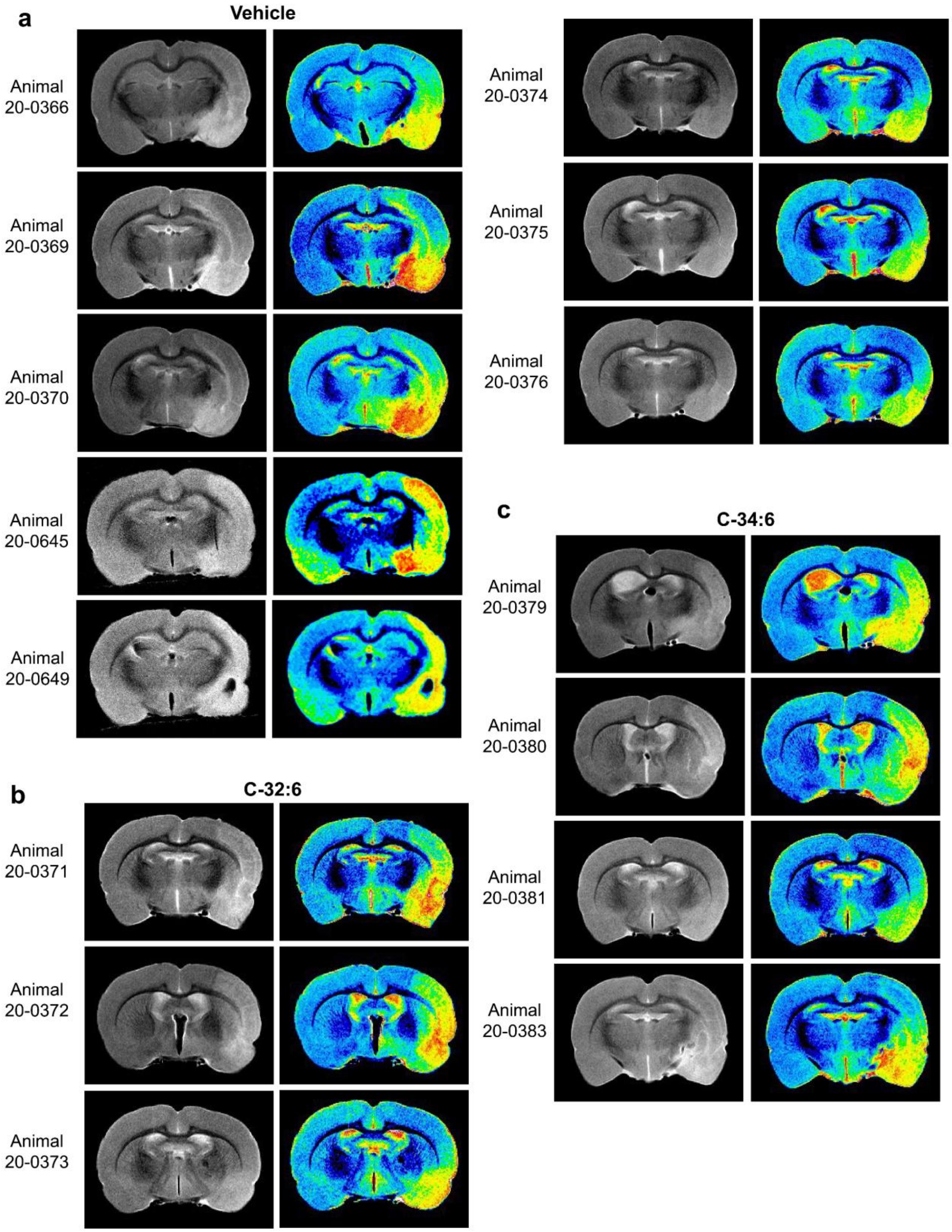

Supplementary Fig. S2: Representative bregma level T2WI and pseudo-colored T2WI images for all animals. Vehicle (**a**), C-32:6 (**b**), and C-34:6 groups (**c**).

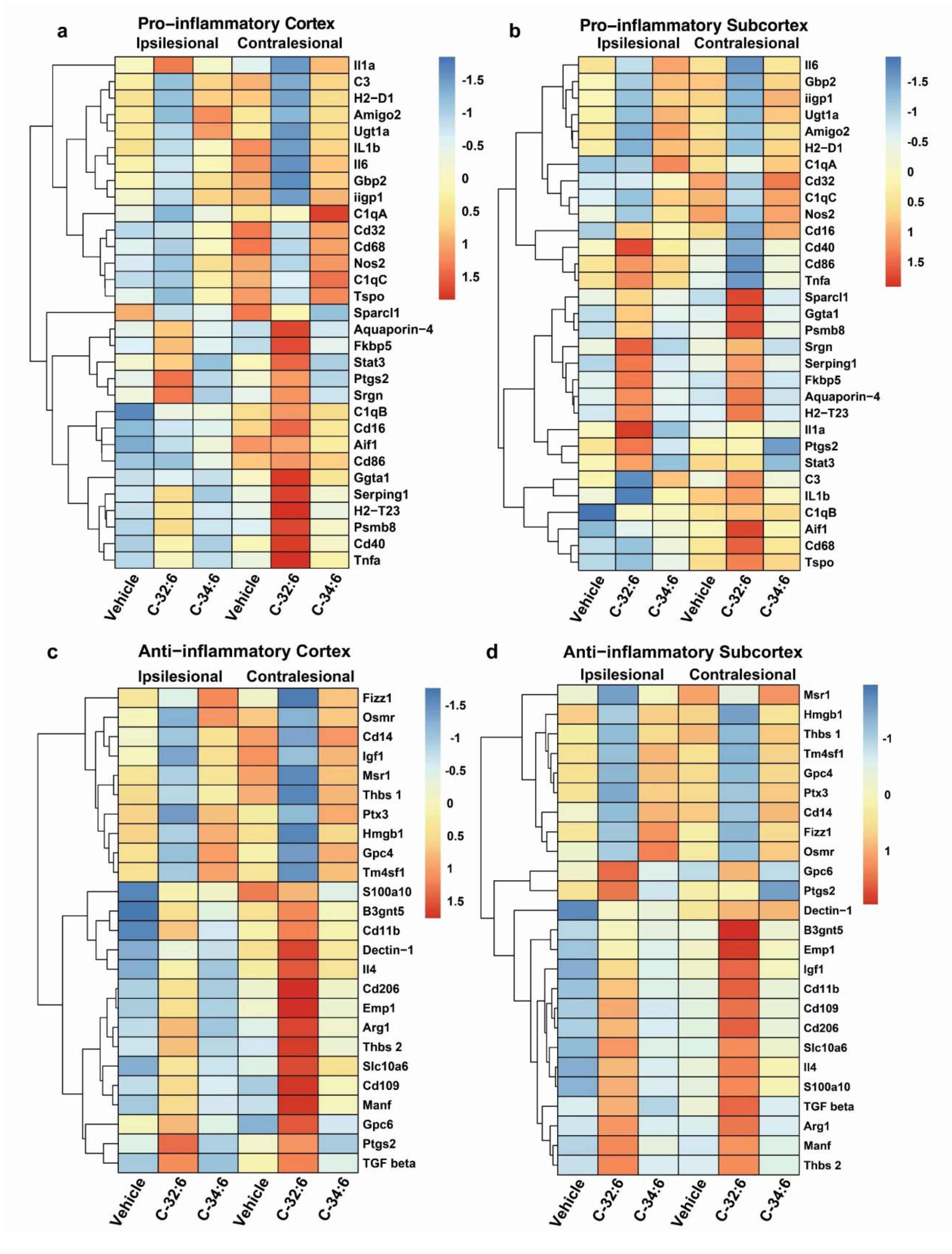

Supplementary Fig. S3: Heatmaps for pro- and anti-inflammatory genes show differential regulation in the ipsi- and contralesional cortex (a, c) and subcortex (b, d).

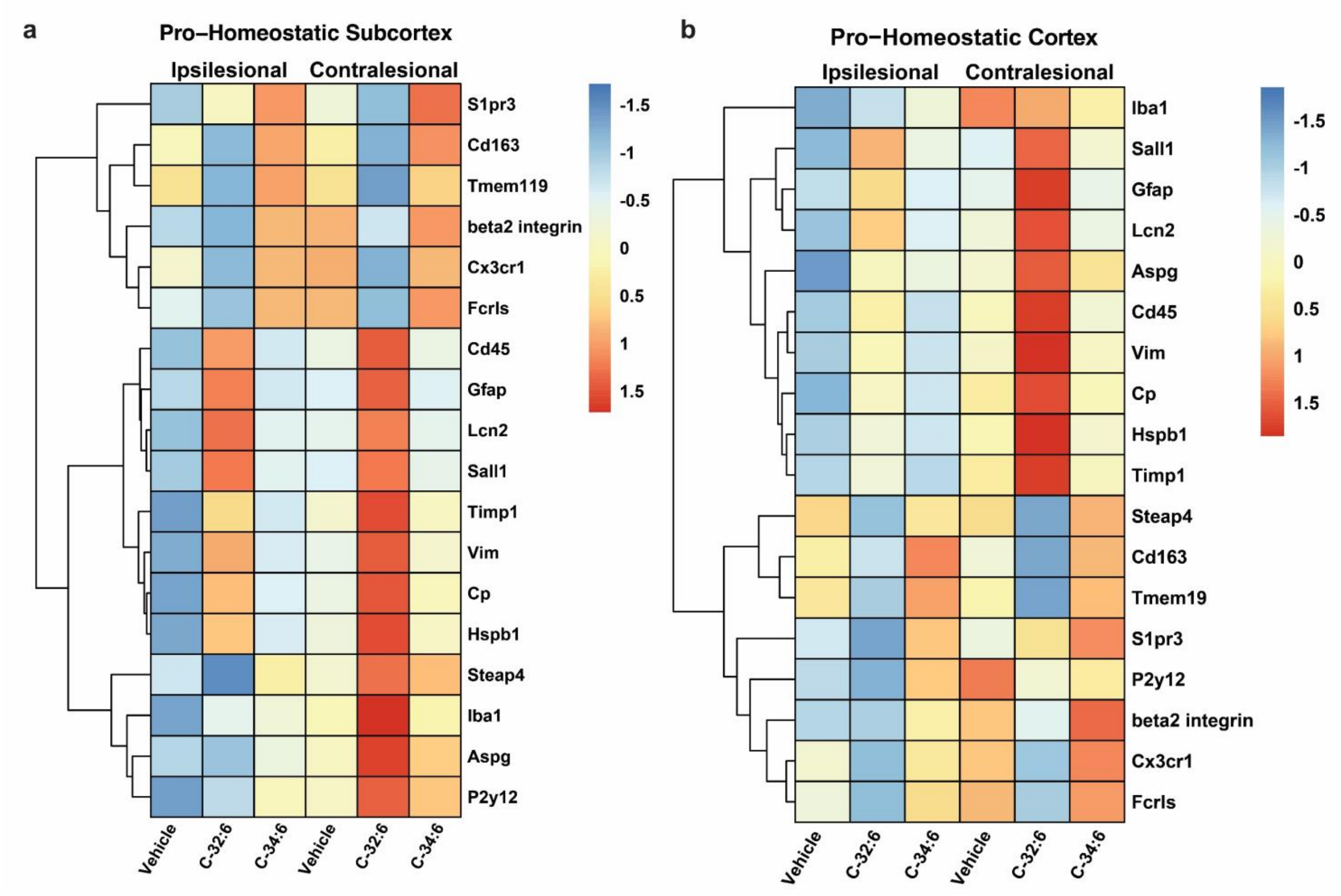

Supplementary Fig. S4: Heatmaps for pro-homeostatic genes showing differential regulation in the ipsi- and contralesional subcortex (a) and cortex (b).

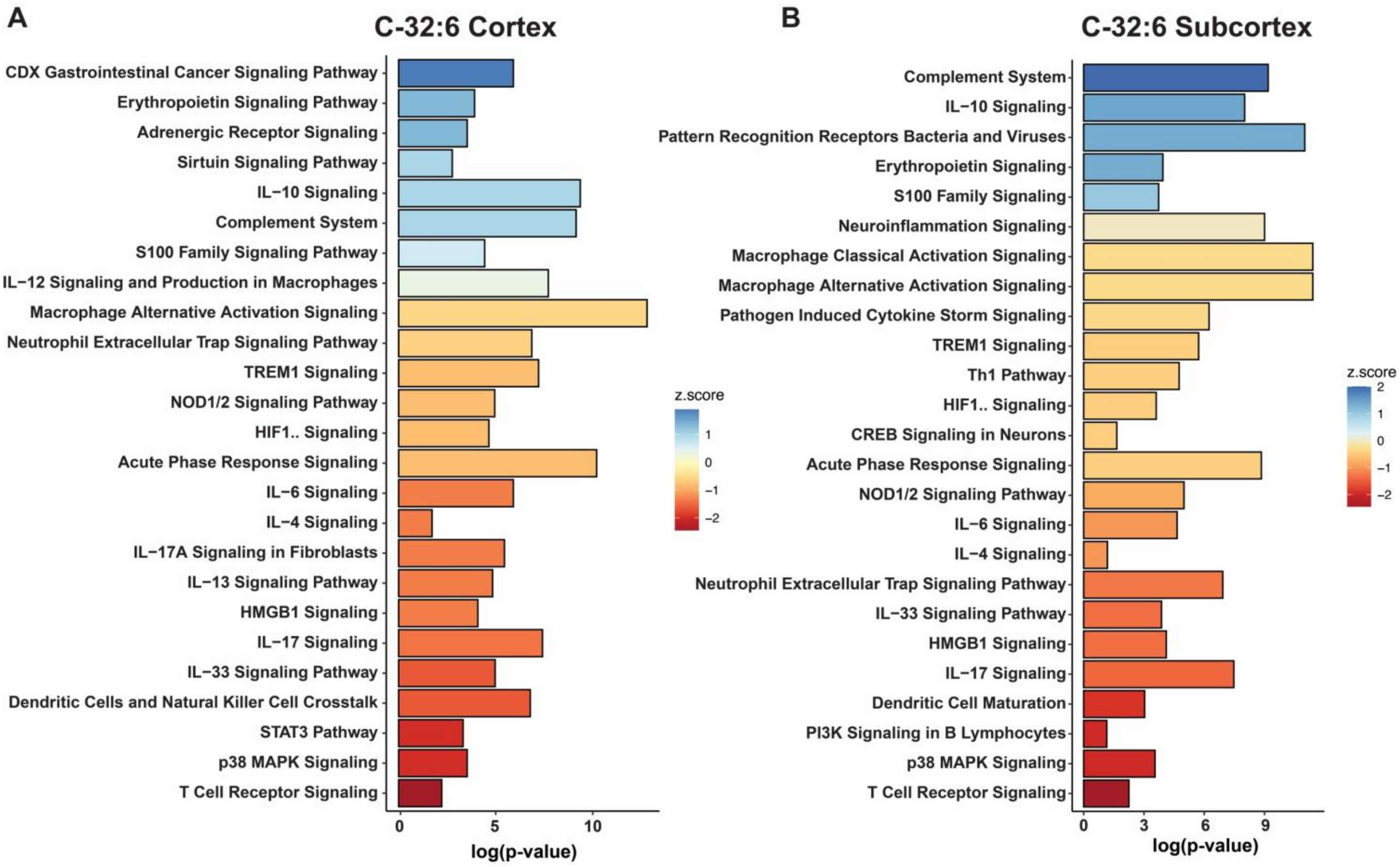

Supplementary Fig. S5: Ingenuity pathway analysis of C-32:6 gene expression in contralesional cortex and subcortex brain regions. a, b, Canonical pathway plots showing p-value and z-score computed from the ingenuity pathway analysis algorithm based on the uploaded gene set in the cortex (**a**) and in the subcortex (**b**).

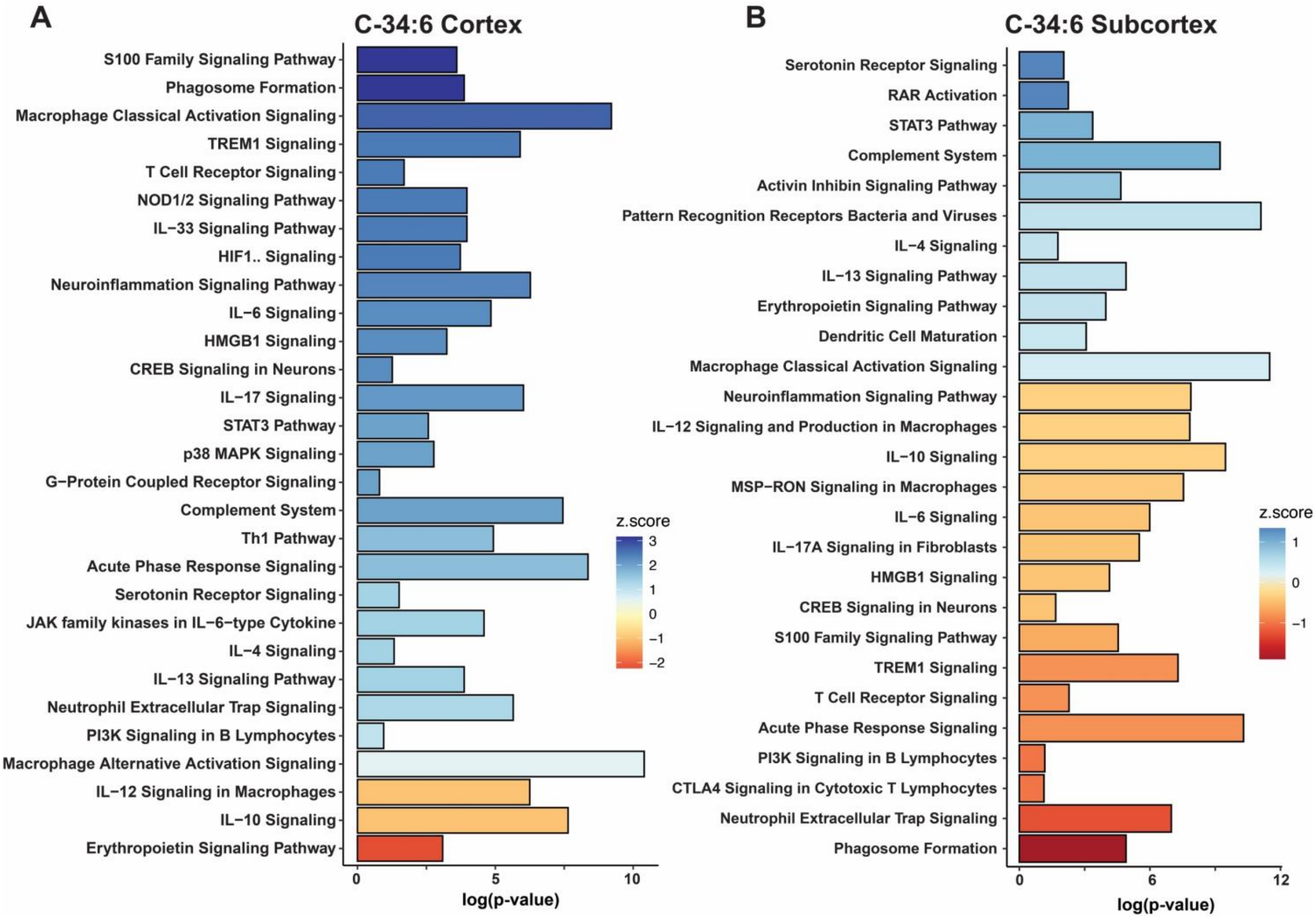

Supplementary Fig. S6: Ingenuity pathway analysis of C-34:6 gene expression in contralesional cortex and subcortex brain regions. a, b, Canonical pathway plots showing p-value and z-score computed from the ingenuity pathway analysis algorithm based on the uploaded gene set in the cortex (**a**) and in the subcortex (**b**).

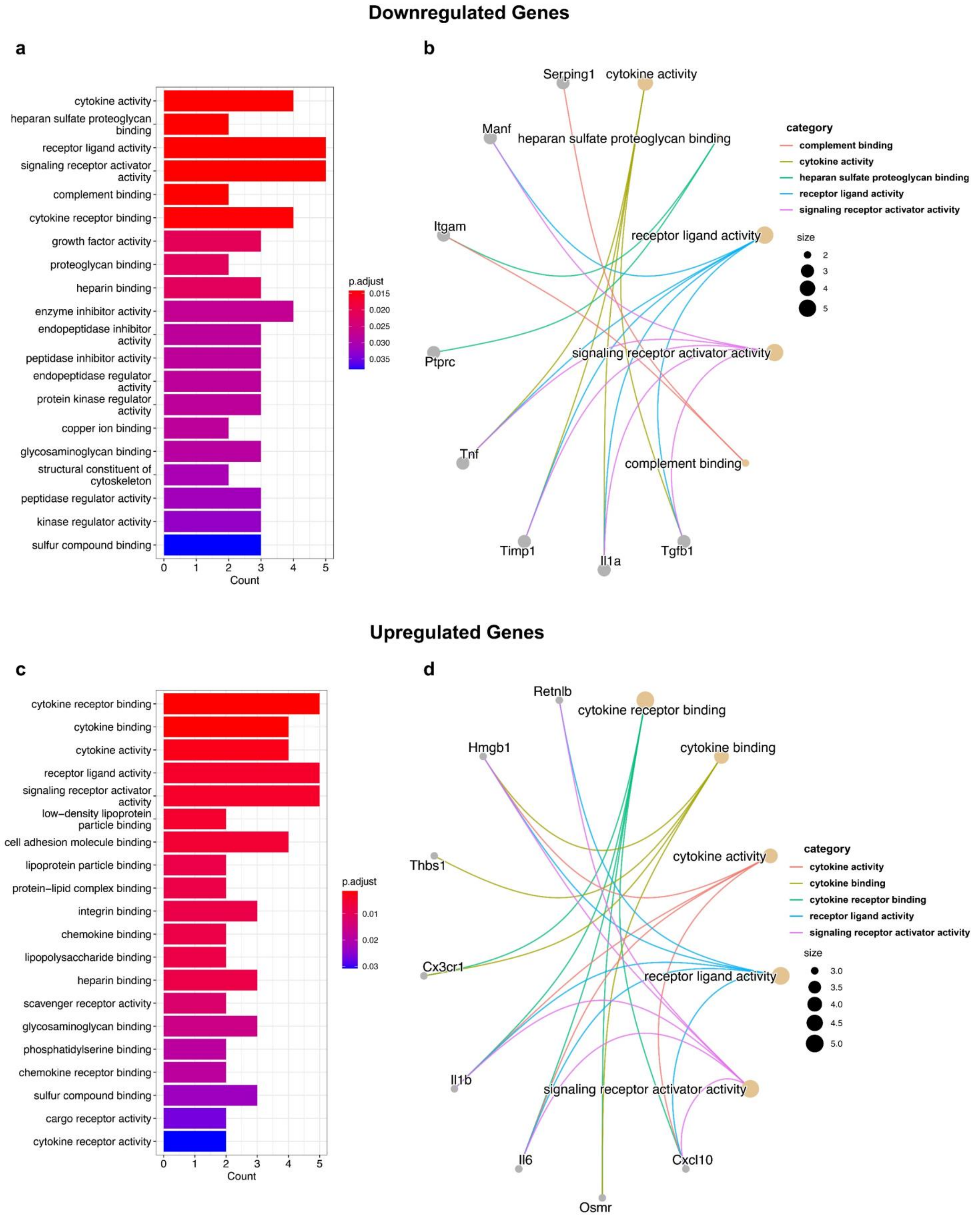

Supplementary Fig. S7: Gene ontology of C-32:6 differentially expressed genes in the subcortex. Barplot showing enriched GO terms from upregulated genes (**a**) cnet plot of genes and the GO terms they are enriched in (**b**). Enrichment barplot from GO analysis of downregulated genes (**c**) network plot of the downregulated genes and their associated terms (**d**).

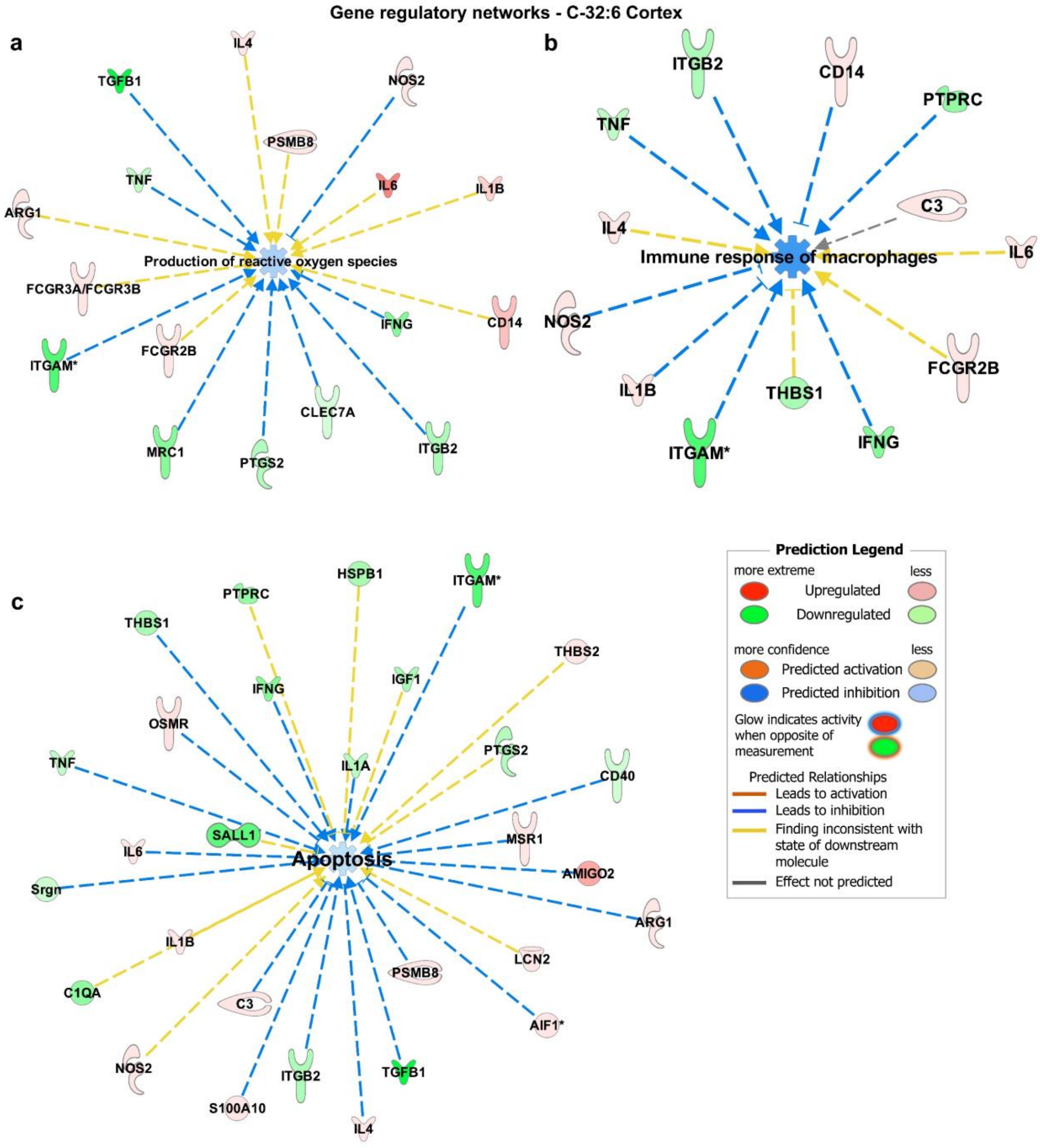

Supplementary Fig. S8: Gene regulatory networks in the cortical region predicted and observed to be modulated by C-32:6. Predicted inhibition of the reactive astrocytes (**a**), immune response of macrophages (**b**), and apoptosis (**c**).

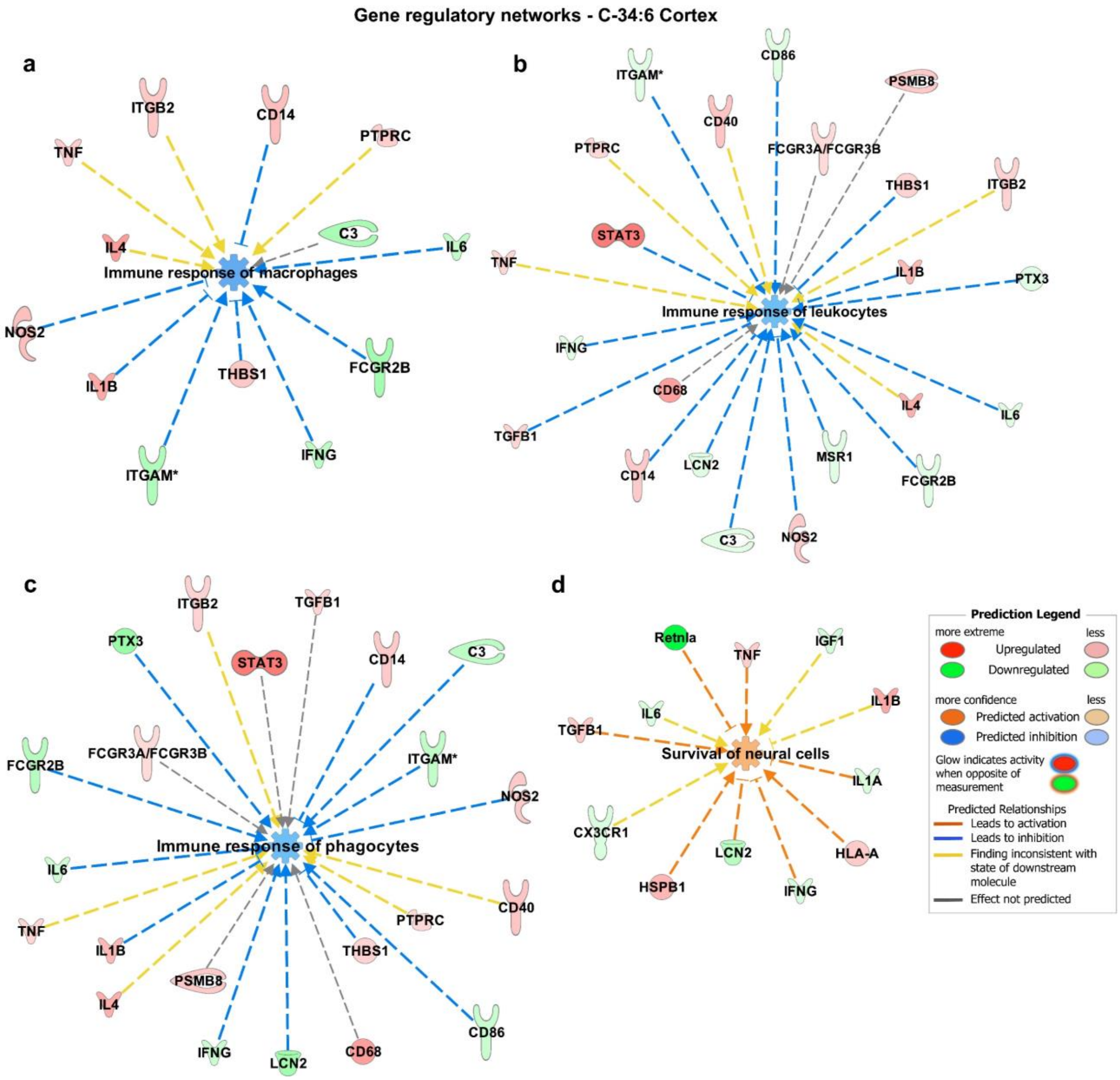

Supplementary Fig. S9: Gene regulatory networks in the cortical region predicted and observed to be modulated by C-34:6. Predicted inhibition of the immune response of macrophages (**a**), immune response of leukocytes (**b**), immune response of phagocytes (**c**), and activation of the survival of neural cells (**d**).

SUPPLEMENTARY TABLES

Supplementary Table S1

		Volumes			
**Groups**	**Animal Number**	**Total brain vol**	**Total Lesion**	**Core**	**Penumbra**
**Saline N = 5**	20-0366	1324.23	153.20	79.99	73.21
	20-0369	1259.24	192.72	89.32	103.40
	20-0370	1393.29	147.44	66.48	80.96
	20-0645	1789.56	86.82	84.83	1.98
	20-0649	1619.32	152.06	109.95	42.11
**Average ± SEM**		**1477.1 ± 98.9**	**146.4 ± 17.0**	**86.1 ± 7.1**	**60.3 ± 17.6**
**C-32:6 N = 6**	20-0371	1316.99	51.74	24.60	27.14
	20-0372	1288.90	45.79	19.95	25.84
	20-0373	1186.59	9.37	4.91	4.47
	20-0374	1267.47	1.50	0.16	1.34
	20-0375	1169.21	2.89	0.51	2.38
	20-0376	1195.55	3.00	1.48	1.53
**Average ± SEM**		**1237.5 ± 25.1**	**19.1 ± 9.5**	**8.6 ± 4.4**	**10.5 ± 5.1**
**C-34:6 N = 4**	20-0379	1208.80	87.70	64.83	22.86
	20-0380	1253.87	69.13	34.78	34.35
	20-0381	1149.11	12.34	3.70	8.64
	20-0383	1035.17	61.77	28.27	33.50
**Average ± SEM**		**1161.7 ± 47.3**	**57.7 ± 16.1**	**32.9 ± 12.6**	**24.8 ± 6.0**

Supplementary Table S1: T2WI MRI computed total, core, and penumbra lesion volumes for all animals in vehicle, C-32:6, and C-34:6 treatment groups.

Supplementary Table S2

Symbol	***Entrez Gene Name***	***Fold Change Ratio***
ARG1	arginase 1	-13.163
FCGR2B	Fc gamma receptor IIb	1.071
IL4	interleukin 4	-11.5
IL6	interleukin 6	10.468
IL1A	interleukin 1 alpha	-51.564
IL1B	interleukin 1 beta	4.575
MRC1	mannose receptor C-type 1	-75.753
NOS2	nitric oxide synthase 2	3.728
Retnla	resistin like alpha	37.101
STAT3	signal transducer and activator of transcription 3	-187.662
TGFB1	transforming growth factor beta 1	-122.85
THBS1	thrombospondin 1	-145.583
TNF	tumor necrosis factor	-26.087

Supplementary Table S2: Gene expression fold change ratio values in C-32:6 samples for genes involved in macrophage activation signaling pathway generated in IPA.

Supplementary Table S3

Symbol	*Entrez Gene Name*	*Fold Change Ratio*
ARG1	arginase 1	1.056
FCGR2B	Fc gamma receptor IIb	-1.733
IL4	interleukin 4	-1.576
IL6	interleukin 6	-2.191
IL1A	interleukin 1 alpha	1.277
IL1B	interleukin 1 beta	-2.056
MRC1	mannose receptor C-type 1	-1.634
NOS2	nitric oxide synthase 2	-1.293
Retnla	resistin like alpha	-8.134
STAT3	signal transducer and activator of transcription 3	2.116
THBS1	thrombospondin 1	-1.334
TNF	tumor necrosis factor	-1.013

Supplementary Table S3: Gene expression fold change ratio values in C-34:6 samples for genes involved in macrophage activation signaling pathway generated in IPA.

Supplementary Table S4

Target or Reference Gene	Primer Sequence	Ensembl ID
AIF1	F- AAGGATTTGCAGGGAGGAAAAGC<br>R- CTCCATGTACTTCGTCTTGAAGG	ENSRNOG00000000853
AMIGO2	F – ACCGACGGCTGGCTAAGTAT<br>R - AGGGTGCATTCTGCCTTAACT	ENSRNOG00000007032
AQUAPORIN-4	F – GGAAGGCATGAGTGACGGA<br>R - GCTTGAGTCCAGACGCCTTT	ENSRNOG00000016043
ARG1	F -GCTTGAGTCCAGACGCCTTT<br>R -CTCCTCGAGGCTGTCCCTTA	ENSRNOG00000013304
ASPG	F- GTACGGAAGGCCAGTTGGAA<br>R - GCCAAAGGTCTCCATGACCA	ENSRNOG00000012843
B3GNT5	F – AGTCTCCGAAGTCACACCCA<br>R - GCACGTCCAAATCCGTTGAC	ENSRNOG00000046258
BETA2 INTEGRIN	F – CCTTCTCTCCACAGGACATGC<br>R - GTGAAGTTCAGCTTCTGGCAC	ENSRNOG00000001224
C1QA	F – AGGTCACTTCATCTGTGCGG<br>R - CCCCTGCTAACACCTGGAAG	ENSRNOG00000012807
C1QB	F – CCTTCTGCGACTATGCCCAA<br>R - CCTGCAGGTGAACAACCTCT	ENSRNOG00000012749
C1QC	F – AGCATCAGTCGGTGTTCACG<br>R - GAGGATTGGTGATGGCGGAA	ENSRNOG00000012804
C3	F – CAAACTTCGGGGCAACAGTG<br>R - TCTTGCCCACAGGCAATAGG	ENSRNOG00000046834
CD109	F – AGGAATCATCAGACCCGGAGC<br>R - GCCGGAAGAACAAGAATTCCG	ENSRNOG00000025332
CD11B	F – CTATTCGGCTCCAACCTGCT<br>R - TCGATCGTGTTGATGCTACCG	ENSRNOG00000019728
CD14	F – ATTGCCCAAGCACACTCACT<br>R - TCAGTCCTTTCTCGCCCAAC	ENSRNOG00000017819
CD16	F – CGTCCATGCAGACTGGCTAT<br>R - TTTCGCCAGCTATGGCATCT	ENSRNOG00000024382
CD163	F – TCCGGTTGAAGTTTTGTGACC<br>R - GTGGTCCCGATGACCGTATT	ENSRNOG00000010253
CD206	F – TGATTCCGGTCGCTGTTCAA<br>R - GAACGGAGATGGCGCTTAGA	ENSRNOG00000018251
CD32	F – AGGTTCCAGACACTCCTTCTG<br>R - CCGGAGCTTCAGGATGCTTG	ENSRNOG00000046452
CD40	F – AGGTGGTCAAGAAACCAAAGGA<br>R - GCAGTGTTGTGACCGGGATA	ENSRNOG00000018488
CD45	F – ACCATCCACTCTGGGCTTTG<br>R - ACGCACAGTAACGTTCCCAA	ENSRNOG00000000655
CD68	F – GCCCTCACCAAGTCCTAGTC<br>R - GCTTGGAGCTGAACACATGG	ENSRNOG00000037563
CD86	F – AGACATGTGTAACCTGCACCAT<br>R - GAGCTCACTCGGGCTTATGT	ENSRNOG00000038835
CP	F – TTCATGCGGGGATGGTAACT<br>R - TGAACAGTTGTGTGGCTTTGA	ENSRNOG00000011913
CX3CR1	F – TCTTCACGTTCGGTCTGGTG<br>R - GGCAAAGTGGCCACAAAGAG	ENSRNOG00000018509
CXCL10	F – ACCCAGGGCCATAGGAAAAC<br>R - CTTTGGCTCACCGCTTTCAA	ENSRNOG00000022256
DECTIN-1	F – AGGAGAACCACAAACCCACAG<br>R - GCAAGGTCCAGAAAAGACGC	ENSRNOG00000054251
EMP1	F – CATCGCCACTGCCATTATGC<br>R - TGCAGTTCTTCCAAAGCCCT	ENSRNOG00000008676

FCRLS	F – GTGCGTTTGGTGAATGGTCC<br>R - CTTCATGTCCCAGCCATCGT	ENSRNOG00000001955
FIZZ1	F – CAGCTGATGGTCCCAGTGAAT<br>R - GGAGGCCCATTTGCTCATAGA	ENSRNOG00000001955
FKBP5	F – CAGAGCAGGATGCCAAGGAA<br>R - TCCCATGGTCTGACTCTCGT	ENSRNOG00000022523
GBP2	F – TACCTGGAGCATTCGCTGAC<br>R - GTTGCTTCCTCAGAGCAGGT	ENSRNOG00000031743
GFAP	F – GAAATTGCTGGAGGGCGAAG<br>R - ATTTGGTGTCCAGGCTGGTT	ENSRNOG00000002919
GGTA1	F – GCTGAAGACCTCACTCTCAGG<br>R - GAGTTCTATGGAGCTCCCGC	ENSRNOG00000019179
GPC4	F – ACTCTGCTCTCCTGCGACG<br>R - GGAAAAGGATCGGTGACCTCG	ENSRNOG00000002413
GPC6	F – ACACCCGCTTTCGACCCTA<br>R - CTGCATGGTCCTCCACACAGT	ENSRNOG00000046660
H2-D1	F – ATGGAACCTTCCAGAAGTGGG<br>R -GAAGTAAGTTGGAGTCGGTGGA	ENSRNOG00000030712
H2-T23	F – ATTGGAGCTGTTGTGAGGAGG<br>R - CCACGAGGCAACTGTCTTTTC	ENSRNOG00000000777
HMGB1	F – GCCCATTTTGGGTCACATGG<br>R - ACCCCTACCACAATATGCAGG	ENSRNOG00000058908
HSPB1	F – GTGGAGATCACTGGCAAGCA<br>R - ACCTGGAGGGAGCGTGTATT	ENSRNOG00000023546
IBA1	F – GCTATGAGCCAGAGCAAGGAT<br>R - GCTTGTTGATCCCATCCAACC	ENSRNOG00000000853
IGF1	F – GTACCAAAATGAGCGCACCTC<br>R - GCTGGTAAAGGTGAGCAAGC	ENSRNOG00000004517
IIGP1	F – ATTTGGCTCGAAGCCTTTGC<br>R - ACGGCATTTGCCAGTCCTTA	ENSRNOG00000038960
IL10	F – CCTCTGGATACAGCTGCGAC<br>R - TGGCCTTGTAGACACCTTTGT	ENSRNOG00000004647
IL1A	F – CGCTTGAGTCGGCAAAGAAA<br>R - AGACAGATGGTCAATGGCAGA	ENSRNOG00000004575
IL1B	F - CCTATGTCTTGCCCGTGGAG<br>R - CACACACTAGCAGGTCGTCA	ENSRNOG00000004649
IL4	F -CCCTGAACTCAACTGTGAAATAGCA<br>R - CCCAAGTCAAGGGCTTGGAA	ENSRNOG00000007624
IL6	F - TCTCCGCAAGAGACTTCCAG<br>R - AAGTCTCCTCTCCGGACTTGT	ENSRNOG00000010278
INF GAMMA	F - CATCGCCAAGTTCGAGGTGA<br>R - CACCGACTCCTTTTCCGCTT	ENSRNOG00000007468
ITGAM	F - GACTCCGCATTTGCCCTACT<br>R - TGCCCACAATGAGTGGTACAG	ENSRNOG00000019728
LCN2	F - ATTGTATGAACAGCGATGATGCAC<br>R - CCAGGTAGAAACGGAACTCCAGA	ENSRNOG00000013973
MSR1	F – TGGCTGAATGAAGTGATGTGC<br>R - CAAGTGACCCCAGCATCTTCT	ENSRNOG00000012779
NOS2	F – CAAGTGGCCGACACTGACTA<br>R - TTCAGTTCATCGGACAGCCC	ENSRNOG00000057443
OSMR	F – TCCCTGGTCGAAAGTCCAAC<br>R - CATGGGTGGGTTCGTGACAA	ENSRNOG00000033192
P2RY12	F – TTGCACGGATTCCCTACACC<br>R - GGGTGCTCTCCTTCACGTAG	ENSRNOG00000013902
PSMB8	F – TGCAGCCTCCAAGCTACTTT<br>R - GCTGCCTGTGGAGAACATCT	ENSRNOG00000000456
PTGS2	F – CTCAGCCATGCAGCAAATCC<br>R - GGGTGGGCTTCAGCAGTAAT	ENSRNOG00000002525

PTX3	F – TATGGCACCAAGTGGAACCC<br>R - GGAGACCACAGTATCCGCAG	ENSRNOG00000012280
S100A10	F – AGGGAGTTCCCTGGGTTTTT<br>R - AAAGCTCTGGAAGCCCACTT	ENSRNOG00000023226
SALL1	F - CATTTCCAATCCGACCCCGA<br>R - CTTTGCTCTTAGTGGGGCGA	ENSRNOG00000013907
SERPING1	F – CAGAGGCTAACTGGCTTCGT<br>R - TGTGGCTGGTCACTTCAGAAT	ENSRNOG00000007457
SLC10A6	F – GCTGTGCATTGCCATGATGC<br>R - GCCTGATATGCTGCGACAATG	ENSRNOG00000002057
S1PR3	F – CTTGCAGAACGAGAGCCTGT<br>R - CCTCAACAGTCCACGAGAGG	ENSRNOG00000014524
SPARCL1	F – GCCGACACTGGAAGATGCTA<br>R - CTGTCGACTGTTCATGGGCT	ENSRNOG00000015093
SRGN	F – AGAAAGGACCACGGTTCGAC<br>R - TGGGGAAGAAATCATTCGGGA	ENSRNOG00000000394
STAT3	F – GACCGCGTCGGCTAGGA<br>R - TGAGCCATCCTGCCGCAAT	ENSRNOG00000019742
STEAP4	F – GGCTCTCCAGTCAGGAACAC<br>R - AAGAGTGCGAGCAATGTCCA	ENSRNOG00000008602
TGF BETA	F – CTGCTGACCCCCACTGATAC<br>R - AGCCCTGTATTCCGTCTCCT	ENSRNOG00000020652
THBS1	F – TAGCTGGAAATGTGGTGCGT<br>R - AGCAAGCATCAGGCACTTCT	ENSRNOG00000045829
THBS2	F – TGGAAGGACTACACCGCCTA<br>R - GAGTCAGCCATGACCTGCTT	ENSRNOG00000010529
TIMP1	F – CTGCAACTCGGACCTGGTTA<br>R - CAGCGTCGAATCCTTTGAGC	ENSRNOG00000010208
TM4SF1	F – TGCTTCCCAGACTTGGTTGT<br>R - TGAGAGAGGGTGTCACTTCAGA	ENSRNOG00000015812
TMEM119	F – GACTCCTCCCTGCTTGCTTTC<br>R - CCAAAACTTCGAGTCACTGCT	ENSRNOG00000000700
TNFA	F – ATGGGCTCCCTCTCATCAGT<br>R - GCTTGGTGGTTTGCTACGAC	ENSRNOG00000070745
TSPO	F – TGTGGATCTTTCCAGAACAGCA<br>R - CGCACAAAGTAGGCTCCCAT	ENSRNOG00000010549
UGT1A	F – GGAAGCTGTTAGTGATCCCC<br>R - TGCTATGACCACCACTTCGT	ENSRNOG00000018740
VIM	F – CATGCGGCTGCGAGAAAAAT<br>R - TTCAAGGTCAAGACGTGCCA	ENSRNOG00000018087
ACTB	F – CCCGCGAGTACAACCTTCT<br>R - CGACGAGCGCAGCGATA	ENSRNOG00000034254
GAPDH	F - GCATCTTCTTGTGCAGTGCC<br>R - GGTAACCAGGCGTCCGATAC	ENSRNOG00000018630
GUSB	F – CAGTTGTGTGGGTGAATGGG<br>R - GGTCAGTGTGTTGTTGATGGC	ENSRNOG00000000913
PGK1	F – AAAGTCAGCCATGTGAGCACT<br>R - CCACTAGCTGCACTAACACC	ENSRNOG00000058249
PP1A	F – GCAGACTTTGCTTTCCTTGG<br>R - CGAGAGGTCCTTTTCACCAG	ENSRNOG00000018708
RPL13A	F – GGATCCCTCCACCCTATGACA<br>R - CTGGTACTTCCACCCGACCTC	ENSRNOG00000020618
TFRC	F – CCGGCCTATATGCTTGGGTA<br>R - CAAGGGAGCACTCTGAAGCA	ENSRNOG00000001766

Supplementary Table S4: Primer table of all target genes measured. Forward and reverse primer sequences and Ensembl gene ID are included.

Supplementary Table S5

Gene	***Fold Change***	***df***	**statistic**	***p***	**95% CI**
Stat3	6.318	8.26	3.11	.014	[0.65, 4.29]
S1pr3	1.081	14.96	-2.56	.022	[-4.75, -0.43]
Tmem119	0.793	13.50	-2.25	.042	[-2.49, -0.06]
Cd163	0.053	8.01	-2.41	.043	[-6.99, -0.15]

Supplementary Table S5: Welch's individual t-test results for ΔCt comparisons between C-34:6 vs. Vehicle in the ipsilesional cortex.

Supplementary Table S6

Gene	*Fold Change*	*df*	*statistic*	*p*	*95% CI*
Steap4	1.659	10.54	3.72	.004	[2.30, 9.05]
TGF	0.205	4.32	-4.06	.013	[-10.84, -2.19]
Ptx3	48.561	3.99	4.11	.015	[1.82, 9.46]
Stat3	0.685	8.85	-2.82	.020	[-3.92, -0.43]
Osmr	11.353	2.95	3.78	.033	[0.34, 4.19]

Supplementary Table S6: Welch's individual t-test results for ΔCt comparisons between C-32:6 vs. Vehicle in the ipsilesional cortex.

Supplementary Table S7

Gene	*Fold*	*df*	statistic	*p*	95% CI
Tm4sf1	57.555	13.29	3.05	.009	[0.45, 2.62]
Ugt1a	128.553	13.72	2.98	.010	[0.73, 4.50]
Tspo	1.472	5.59	3.30	.018	[0.63, 4.48]
S1pr3	0.332	14.00	2.59	.021	[0.48, 5.05]
Stat3	0.332	7.44	-2.76	.026	[-4.24, -0.36]
Amigo2	39.997	13.74	2.46	.028	[0.18, 2.71]
Fcrls	2.660	5.32	2.97	.029	[0.49, 6.09]
Cd11b	0.278	5.20	2.97	.030	[0.28, 3.65]
C1qC	1.203	4.78	2.91	.035	[0.19, 3.46]
Gbp2	5.412	5.19	2.80	.036	[0.13, 2.58]
beta2 integrin	0.525	4.92	2.81	.038	[0.17, 4.00]
Vim	0.541	4.46	2.89	.039	[0.17, 4.26]
Il4	1.578	5.11	2.66	.044	[0.07, 3.46]
Il1a	0.584	11.98	-2.21	.047	[-4.35, -0.03]

Supplementary Table S7: Welch's individual t-test results for ΔCt comparisons between C-34:6 vs. Vehicle in the ipsilesional subcortex.

Supplementary Table S8

Gene	*Fold Change*	*df*	statistic	*p*	95% CI
Gpc4	12.468	12.06	-7.29	< .001	[-6.03, -3.25]
Tmem119	12.987	11.66	-7.14	< .001	[-5.25, -2.79]
Ugt1a	128.553	12.91	-6.70	< .001	[-7.33, -3.75]
Tm4sf1	57.555	9.90	-7.59	< .001	[-6.14, -3.35]
Msr1	11.193	12.58	-6.51	< .001	[-6.94, -3.47]
Amigo2	39.997	12.30	-5.86	< .001	[-5.08, -2.33]
H2-D1	16.535	9.45	-6.32	< .001	[-5.47, -2.60]
H2-T23	0.475	4.30	10.84	< .001	[3.71, 6.18]
Ptx3	48.561	12.78	-4.89	< .001	[-8.67, -3.35]
Cd163	393.741	9.89	-5.22	< .001	[-6.90, -2.77]
Gfap	0.266	3.95	9.97	.001	[4.84, 8.60]
Sall1	0.280	7.87	5.50	.001	[5.24, 12.84]
C3	6.217	5.71	-6.62	.001	[-6.08, -2.77]
Thbs 1	11.151	5.51	-6.50	.001	[-5.23, -2.32]
Cd86	1.382	7.35	4.94	.001	[1.47, 4.13]
Cxcl10	10.753	5.46	-5.87	.002	[-6.24, -2.51]
Lcn2	1.366	5.39	5.91	.002	[2.43, 6.03]
Nos2	1.534	5.67	-5.68	.002	[-3.31, -1.30]
Osmr	11.353	9.85	-4.11	.002	[-3.49, -1.03]
Cd11b	0.278	3.62	7.63	.002	[3.70, 8.23]
Il6	9.819	4.56	-6.05	.002	[-5.02, -1.97]
Cd206	0.502	3.79	7.06	.003	[3.35, 7.85]
iigp1	4.471	4.56	-5.96	.003	[-3.63, -1.40]
Cd14	5.386	4.28	-6.21	.003	[-3.76, -1.48]
Il1a	0.584	12.04	3.67	.003	[1.42, 5.56]
Hmgb1	4.034	4.26	-5.94	.003	[-3.17, -1.18]
Fkbp5	0.455	3.39	7.38	.003	[3.41, 8.05]
Cp	1.351	3.70	6.29	.004	[3.16, 8.47]
Gpc6	0.502	4.22	5.55	.004	[2.04, 5.96]
Cd45	0.441	3.28	6.75	.005	[4.06, 10.69]
Hspb1	0.581	3.28	6.25	.006	[2.90, 8.39]
Igf1	0.589	2.68	7.84	.006	[1.93, 4.90]
Vim	0.541	3.13	5.79	.009	[3.34, 11.09]
Timp1	0.775	3.18	5.70	.009	[2.69, 9.03]
Manf	0.274	3.74	4.78	.010	[2.81, 11.10]
Tspo	1.472	8.01	-3.14	.014	[-2.55, -0.39]

Fcrls	2.660	11.56	-2.83	.016	[-2.61, -0.33]
C1qB	1.050	4.03	3.90	.017	[0.42, 2.46]
Aquaporin-4	0.532	3.46	4.15	.019	[1.30, 7.75]
Fizz1	48.314	7.50	-2.90	.021	[-7.85, -0.85]
Cd40	0.960	3.18	4.22	.022	[1.39, 8.88]
Serping1	0.960	3.49	3.95	.022	[1.31, 8.94]
Slc10a6	0.566	3.37	3.94	.023	[1.06, 7.75]
Ggta1	0.487	2.37	5.24	.024	[1.23, 7.23]
Arg1	0.487	3.28	3.80	.027	[0.93, 8.25]
Cx3cr1	1.537	4.84	-3.12	.028	[-2.36, -0.21]
Cd16	1.290	3.33	3.60	.031	[0.48, 5.40]

Supplementary Table S8: Welch's individual t-test results for ΔCt comparisons between C-32:6 vs. Vehicle in the ipsilesional subcortex.

Supplementary Table S9

**Area**	***n***	**mean**	***SE***	***df***	***t***	***p***	**95% CI**
Total	6	19.05	19.46	6.39	6.55	< .001	[80.47, 174.33]
Core	6	8.60	8.35	6.89	9.29	< .001	[57.71, 97.32]
Penumbra	6	10.45	18.31	4.67	2.72	.045	[1.82, 97.95]

Supplementary Table S9: Statical testing results for T2WI MRI lesion volume computed from individual Welch's t-test for C-32:6 vs. vehicle.

Supplementary Table S10

**Area**	***n***	**mean**	***SE***	***df***	***t***	***p***	**95% CI**
Total	4	57.73	23.40	6.95	3.79	.007	[33.30, 144.13]
Core	4	32.90	14.43	4.84	3.69	.015	[15.75, 90.69]
Penumbra	4	24.84	18.58	4.90	1.91	.116	[-12.57, 83.56]

Supplementary Table S10: Statical testing results for T2WI MRI lesion volume computed from individual Welch's t-test for C-34:6 vs. vehicle.

Supplementary Table S11

**Time After MCAo**	***n***	**test- statistic**	***p***
60min	7	72.50	.491
1	7	133.00	< .001
2	7	132.50	< .001
3	7	133.00	< .001
7	7	133.00	< .001

Supplementary Table S11: Results from individual Wilcoxon Mann Whitney comparison of C-32:6 vs. Vehicle Neurologic data.

Supplementary Table S12

Time After MCAo	*n*	test- statistic	*p*
60min	6	54	.640
1	6	112	< .001
2	6	112	< .001
3	6	110	< .001
7	6	111	< .001

Supplementary Table S12: Results from individual Wilcoxon Mann Whitney comparison of C-34:6 vs. Vehicle Neurologic data.
